# Pharmacogenetic landscape of pain management variants among Mediterranean populations

**DOI:** 10.3389/fphar.2024.1380613

**Published:** 2024-05-15

**Authors:** Haifa Jmel, Wided Boukhalfa, Ismail Gouiza, Roua Ouled Seghaier, Hamza Dallali, Rym Kefi

**Affiliations:** ^1^ Laboratory of Biomedical Genomics and Oncogenetics, Institut Pasteur de Tunis, Tunis, Tunisia; ^2^ Genetic Typing Service, Institut Pasteur de Tunis, Tunis, Tunisia; ^3^ University of Tunis El Manar, Tunis, Tunisia; ^4^ Faculty of Medicine of Tunis, Tunis, Tunisia; ^5^ MitoLab Team, Unité MitoVasc, Unité Mixte de Recherche Centre National de la Recherche Scientifique, Institut National de la Santé et de la Recherche Médicale U1083, SFR ICAT, University of Angers, Angers, France

**Keywords:** pharmacogenes, opioids, Mediterranean populations, adverse drug reaction, admixture

## Abstract

**Background:**

Chronic pain is a major socioeconomic burden in the Mediterranean region. However, we noticed an under-representation of these populations in the pharmacogenetics of pain management studies. In this context, we aimed 1) to decipher the pharmacogenetic variant landscape among Mediterranean populations compared to worldwide populations in order to identify therapeutic biomarkers for personalized pain management and 2) to better understand the biological process of pain management through *in silico* investigation of pharmacogenes pathways.

**Materials and Methods:**

We collected genes and variants implicated in pain response using the Prisma guidelines from literature and PharmGK database. Next, we extracted these genes from genotyping data of 829 individuals. Then, we determined the variant distribution among the studied populations using multivariate (MDS) and admixture analysis with R and STRUCTURE software. We conducted a Chi2 test to compare the interethnic frequencies of the identified variants. We used SNPinfo web server, miRdSNP database to identify miRNA-binding sites. In addition, we investigated the functions of the identified genes and variants using pathway enrichment analysis and annotation tools. Finally, we performed docking analysis to assess the impact of variations on drug interactions.

**Results:**

We identified 63 variants implicated in pain management. MDS analysis revealed that Mediterranean populations are genetically similar to Mexican populations and divergent from other populations. STRUCTURE analysis showed that Mediterranean populations are mainly composed of European ancestry. We highlighted differences in the minor allele frequencies of three variants (rs633, rs4680, and rs165728) located in the *COMT* gene. Moreover, variant annotation revealed ten variants with potential miRNA-binding sites. Finally, protein structure and docking analysis revealed that two missense variants (rs4680 and rs6267) induced a decrease in COMT protein activity and affinity for dopamine.

**Conclusion:**

Our findings revealed that Mediterranean populations diverge from other ethnic groups. Furthermore, we emphasize the importance of pain-related pathways and miRNAs to better implement these markers as predictors of analgesic responses in the Mediterranean region.

## Introduction

According to the International Association for the Study of Pain (IASP), pain is defined as an unpleasant sensory and emotional experience connected with actual or potential tissue damage, or described in terms of such damage (Raja et al., 2020). Excessive pain can significantly increase psychological health problems and affects the quality of life (Schuh et al., 2020). Pain includes several types such as neuropathic, nociceptive and nociplastic depending on its neuropsychological cause and duration (Isagulyan et al., 2022). It could be divided into two groups: acute and chronic. Chronic pain represents the most frequent condition (Raffaeli et al., 2021). More than 50% of hospitalized patients suffer from chronic pain in Spain and United Kingdom (Fayaz et al., 2016; Muñoz-Alvaredo et al., 2020) and 14% have moderate-to-severe disabling chronic pain (Dueñas et al., 2015; Yoshida et al., 2018; Mills et al., 2019). Therefore, an appropriate pain management by analgesics should be made. The most widely used analgesic class includes opioids, which are derived from natural compounds contained in opium poppy plants (Pathan and Williams, 2012). Opioids exert several effects in the central nervous system, including pain alleviation. They are commonly employed in a variety of therapeutic contexts. However, opioids could be responsible for sever adverse drug reactions (ADR) such as respiratory depression and tolerance. Therefore, it is important to conduct a tailored opioids medication to enhance patient’s quality of life and reduce the risk of induced ADR. There are various opium derivatives such as codeine, tramadol, fentanyl, sufentanil, alfentanil, and remifentanil (Kumar et al., 2019; Lambert, 2020). Inappropriate opioids use can induce moderate-to-severe ADR. For instance, opioid therapy can cause moderate to severe side effects in 20%–30% of patients such as respiratory depression and hypotension, and bradycardia in 11% of patients. These ADR are mainly due to the high interindividual opioid response variability. Therefore, individualized pain management for each patient is important for alleviating patient suffering and morbidity. Furthermore, tailoring the individual’s treatment can improve analgesic outcomes by maximizing efficacy and minimizing toxicities (Mahajan, 2014; Schuh et al., 2020). Interindividual diversity in pain management therapies is mainly driven by behavior, expectations, gender, renal function, psychosocial distress, ethnic origin and genetic variability (Molanaei et al., 2010). Hence, understanding how a patient’s background affects his response to painkillers is essential for developing personalized pain management therapies (Yoshida et al., 2018; Angus and Chang, 2021). The Mediterranean populations comprise regions around the Mediterranean basin including Southern European, Middle Eastern, and North African regions (de Larramendi et al., 2020). Currently clinical practices used for care pain management in these regions include painkiller drug administration, physical therapy and supplementary treatments (including massage and acupuncture). Painkiller prescription in Mediterranean region is based on governmental Survey and standardized guidelines (Polo-Santos et al., 2021). Nonetheless, there are a number of shortcomings in pain management strategies across the Mediterranean region. Current clinical guidelines often prescribe a straightforward drug regimen for pain management of different categories of patients. This could lead to serious pain persistence. Therefore, it is important to develop more specialized guidelines for chronic pain conditions taking into consideration patients’ intrinsic factor (age, sex, disease stage and the presence of other comorbidities) as well as its environmental factors (socioeconomic status and dietary habits). Moreover, primary care physicians (PCPs) often prescribe opioids as first line therapy which could lead to serious adverse drug reactions and drug addiction in some cases. This could be due to the absence of proper pain treatment policy and trainings for PCPs. Therefore, the lack of specialized pain clinics or trained healthcare providers could lead to serious socioeconomic burden in the region (Louriz et al., 2016; Hamdan and Mosleh, 2024). In the other hand, there are considerable evidences highlighting the role of genetic, epigenetic and ethnicity as pivotal factors on pain differences and management. Previous studies have demonstrated that genetic factors influence the individual response to opioids by 24%–60%. In addition, polymorphisms in transporters, receptors, drug-metabolizing enzymes, and other drug targets influences drug efficacy and toxicity. Thus, Pharmacogenomics studies suggest that an effective pain management strategy should take into account the patient’s genetic background and environmental adaptations, in order to maximize effectiveness and help mitigate impairment (Nahin, 2015). Despite the high prevalence of chronic pain in the Mediterranean populations and its socio-economic burden, only few studies have covered the pharmacogenetic of pain management in these populations (Dueñas et al., 2015). Indeed, several studies have reported a high incidence and prevalence of pain in European and North African countries of the Mediterranean region (Miró et al., 2007; Langley, 2011; Ahangar-Sirous et al., 2023). In addition, the incidence of chronic pain in the Mediterranean region was higher than those described in East Asia and American countries (Zimmer et al., 2022). Moreover, these regions are characterized by a high prevalence of drug dependence, especially opioids, compared to other regions. This suggests the presence of an ethnic disparity linked to the pharmacogenetic component in these populations (Amirkafi et al., 2023).

Taking into account all these elements, more pharmacogenetic studies and testing could revolutionize the field of pain management in the Mediterranean region through personalizing the disease treatment depending on the patient’s DNA profile (Kaye et al., 2019). Pharmacogenes that influence treatment outcomes can generally be split into two categories. On the one hand, pharmacodynamics-influencing genes, based on differences in drug target receptors and downstream signal transduction such as the opioid receptor; OPRM1, the enzyme catecholamine methyltransferase, COMT, etc. Pharmacokinetics genes, on the other hand, impact the cytochrome P450 family, enzymes involved in glucuronidation, and drug transporter proteins. Therefore, a better understanding of the biological pathways involved in the pain management process could help develop new therapeutic strategies. Furthermore, a number of genetic variations implicated in analgesics response variability can influence microRNA (miRNA) binding sites. MiRNAs have a crucial role as epigenetic markers in pain physiopathology, since they influence gene expression regulation and RNA silencing. They have the ability to control many target genes and metabolic activities. Indeed, several studies have documented alterations in miRNA expression in migraines, musculoskeletal diseases, and other pain conditions (Dayer et al., 2019; Polli et al., 2020). Furthermore, a single miRNA can regulate several target genes and metabolic processes that are involved in pain processing (Sabina et al., 2022). Determining the regulatory functions of miRNAs in intricate networks that control biological processes requires an understanding of the interactions between miRNAs and their targets. Since there are a large number of potential targets and a vast and increasing number of miRNA species, it is not possible to manually anticipate how a miRNA will interact with its target. Therefore, prediction databases are commonly used *in silico* analysis to determine potential target genes of miRNAs (Liu, Li, and Cairns 2014). These miRNAs can be used as biomarkers of therapeutic efficacy (Dai et al., 2018; Tramullas et al., 2018; Sabina et al., 2022).

In this purpose, our study aims 1) to decipher the pharmacogenetic landscape of chronic pain among Mediterranean populations compared to worldwide populations in order to identify therapeutic biomarkers for personalized pain management and 2) to better understand the biological process of pain management through *in silico* investigation of pharmacogenes pathways.

## Materials and methods

**FIGURE 1:**
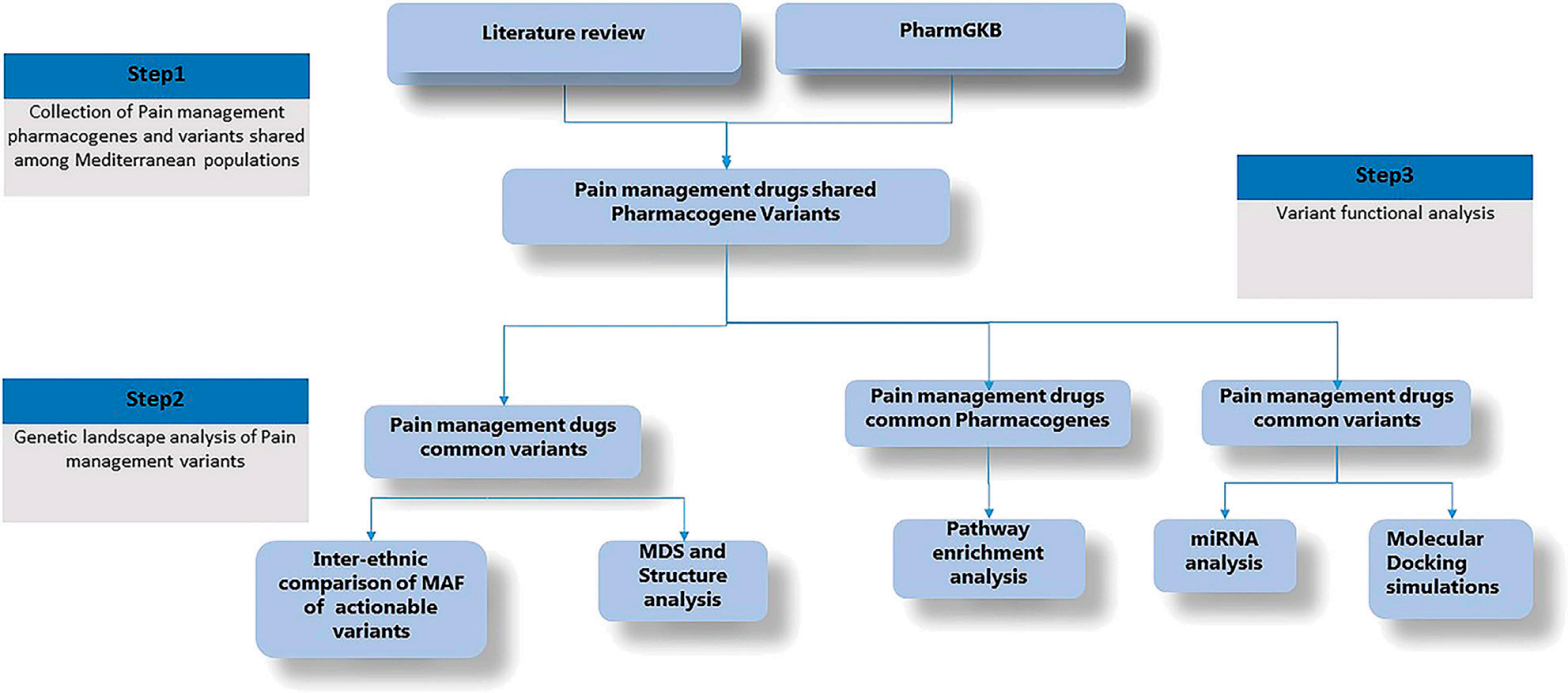
Detailled research workflow. Minor Allele Frequency (MAF), Multidimensional Scaling (MDS), microRNA (miRNA).

To attend our objectives, we developed the present workflow (Figure 1). First, we conducted a general review of the literature to collect pharmacogenes and variants implicated in pain management. Second, we conducted a multidimensional scaling plot (MDS) and Structure analysis of these variants using publicly available genotyping data in order to explore the genetic landscape of pain management among Mediterranean populations compared to other populations. In the third step, we performed *in silico* pathway enrichment, miRNA analysis and simulation docking analysis.

### Step 1: Collection of Pain management pharmacogenes and variants shared among Mediterranean populations

#### Pain management pharmacogene selection

**FIGURE 2:**
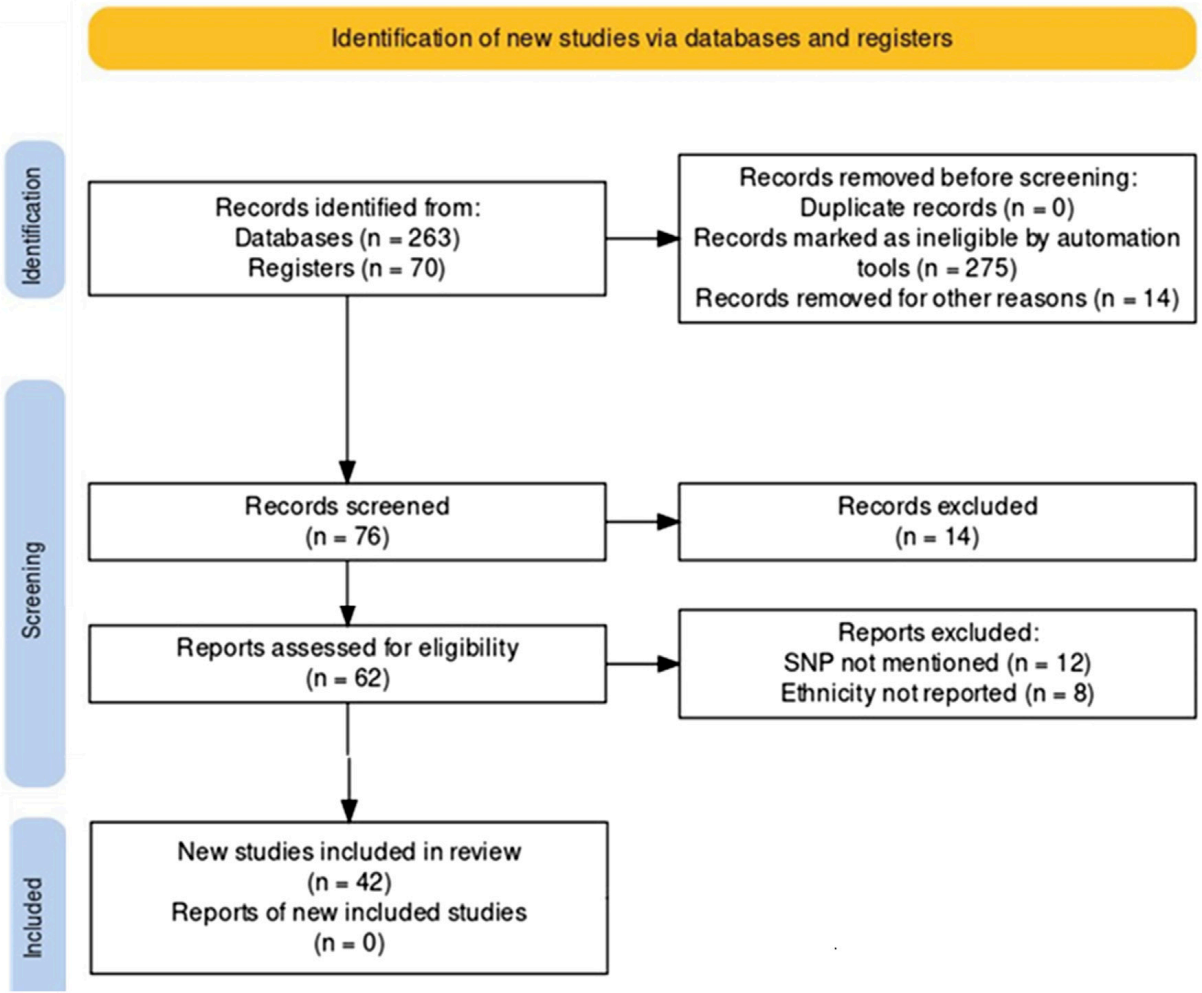
Prisma Flow diagramme of selected studies.

We conducted a systematic search of accessible, peer-reviewed, and complete articles published between January 2000 and April 2023 using the PRISMA guidelines (Page et al., 2021). Articles for review were selected from the MEDLINE (PubMed), Web of Science, and Google Scholar databases. Search terms included a combination of ‘pain management, pain pharmacogenomics of opioids, predictive genetic testing’. This search concerns chronic pain management. The full text version of the included article has been retrieved. In addition, reference lists of relevant studies were evaluated and cheeked by two assessors. Inclusion and exclusion criteria were documented according to the PRISMA rules and presented as a PRISMA flowchart (Figure 2). First, duplicate citations were removed from endnotes, then inappropriate citations linked to missing genotyping data, no ethnic reports, were removed after reading the full articles. Inclusion criteria were papers reporting polymorphisms associated with variation in analgesic response and studies presenting original data with clear and concise endpoint results. Exclusion criteria were non-English studies, abstract-only entries, papers without relevant topics, and studies containing no useful or overlapping data with previously published studies. Several genetic variants were identified according to the Prisma performance criteria. Clinical annotation of these variants was examined using the Pharmacogenomics Knowledge Base, « PharmGKB » http://www.pharmgkb.org, an interactive tool to investigate how genetic variation affects drug response. It displays genotype, molecular, and clinical knowledge integrated into pathway representations and Very Important Pharmacogene (VIP) summaries with links to additional external resources.

### Step 2: Genetic landscape of pain management variants

#### Genotyping data and quality control analysis

We extracted variants located in the selected pharmacogenes using the PLINK v2 toolkit (Chang et al., 2015). Genotypic data of 829 individuals from 16 populations were downloaded from the International 1,000 Genome Project phase III (www.internationalgenome.org) and published data (Li et al., 2008; Henn et al., 2012). The studied populations included those of Mediterranean ancestry: Toscani people of Italy (TSI), South Spain (Spain_S), North Spain (Spain_N), North West of Spain (Spain_NW) and Spain Basic populations (Spain_BASC), Northwestern and Western European ancestry populations of Utah from the CEPH collection (CEU), Algeria (Algeria), Egypt (Egypt), Libya (Libya), Tunisia Douiret (TN_Ber), South Morocco (Morocco_S), North Morocco (Morocco_N), American ancestry: African ancestry in the South Western United States of America (ASW) and people of Mexican ancestry living in Los Angeles, California, United States of America (MEX), individuals from East Asian ancestry: Han Chinese in Beijing, China (CHB), the Chinese population of metropolitan Denver, Colorado, United States of America (CHD) and Japanese in Tokyo, Japan; (JPT) (Supplementary Table S1).

PLINK v2 software was used to manage genomic data. We excluded variants deviating from the Hardy-Weinberg equilibrium (HWE) with a *p*-value of 10^–4^ and had missed genotyping rate ≤95% for each studied populations.

#### Statistical analysis

Merged genotyping data were pruned on the basis of physical distances and linkage disequilibrium (LD) between variants. High-density markers that do not provide additional information were excluded. Next, we used pruning data to study the landscape of the pain management pharmacogene by creating a multidimensional scaling plot (MDS).

To this end, a symmetric matrix of the identity-by-state (IBS) distances for all pairs of individuals was performed based on the proportion of shared common alleles. This analysis was performed using Plink (Chang et al., 2015) and R software (https://www.R-project.org/).

In addition, allele frequencies of druggable pharmacogene variants selected from common and rare shared variants were calculated. At this stage, the study populations were clustered according to their geographical origin. Three groups were formed; Mediterranean (MED), East Asian (EAS), and American (AMR) populations. A chi-square test was used to compare the allele frequencies of the selected variants between the MED populations and the other populations. Bonferroni’s adjustment was applied to the significance level and set to a *p*-value threshold of 5% divided by the number of tested variants. All analyzes were performed using R software.

#### Analysis of population genetic structures

The STRUCTURE ver. 2.3.4 software (Pritchard, Stephens, and Donnelly 2000; Falush, Stephens, and Pritchard 2007) was used to assess the population structure. This program implements a model-based clustering method to infer population structure using genotype data of unlinked markers. We used the admixture model and correlated allele frequency version of STRUCTURE. The Markov Chain Monte Carlo iteration for each structure analysis was run for 10,000 after an initial burn-in period of 10,000 steps. We calculated delta K as proposed by Evanno et al. (Evanno et al., 2005) to assess the most likely numbers of clusters. Next, we evaluate the similarity of the runs at each K level with the CLUMPAK software (Kopelman et al., 2015). We used the Distruct software in order to illustrate the optimal alignment of subpopulations inferring population substructure and individual assignment across the best runs at each k level (Rosenberg, 2004).

### Step 3: Variant functional analysis

#### Pathway enrichment analysis

We have conducted a pathway enrichment analysis (PEA) to get a deeper insight on the biological functions and pathways involved in pain management. Gene Ontology (GO), Kyoto Encyclopedia of Genes and Genomes (KEGG), WikiPathway (UP_KW), InterPro pathway, SMART pathway, BioCarta Pathways and the Biological Biochemical Image Database (BBID) were investigated for functional annotation and enrichment analysis. A total of 381 pathways with minimum gene set size ≥2 were analyzed using Database for Annotation, Visualization and Integrated Discovery (DAVID), a web-based gene-enrichment analysis tool (https://david.ncifcrf.gov/). We have set a *p*-value = 0.05 as a significant threshold after multiple corrections. GO terms were then visualized using the Monash Gene Ontology (MonaGO) (https://monago.erc.monash.edu/) (Xin et al., 2022). It provides an intuitive, interactive and responsive interface for performing GO enrichment analysis and result visualization (Xin et al., 2022).

#### MiRNAs analysis

MiRNAs play a crucial role as pain efficacy indicators. To this end, we explored the SNPinfo web server to detect variants with potential miRNA-binding sites (Xu and Taylor, 2009). Next, we used miRdSNP database (Bruno et al., 2012) to identify the miRNAs of interest. Finally, the obtained list of miRNAs was explored to generate a heat map of GO pathways affected by this miRNA using miRPathD.

#### Structural effect of the selected variants

To investigate the effects of the identified Single Nucleotide Polymorphisms (SNP) on protein structure, we initially retrieved the 3D crystallized proteins from the RCSB database (https://www.rcsb.org/). Mutant proteins were created using UCSF Chimera 1.17.1 software, followed by an energy minimization step. We employed 1,000 steepest descent steps with a root mean square gradient of 0.02, using the Amber ff12SB force field. The energy minimization involved an update interval of 10 (Yousuf et al., 2017).

To compare the wild-type and mutant structures, we calculated the Root Mean Square Deviation (RMSD) using Chimera software. To assess the potential impact of the identified SNPs on protein stability, we used the mCSM (http://biosig.unimelb.edu.au/mcsm/stability), SDM (http://marid.bioc.cam.ac.uk/sdm2/), DUET (http://biosig.unimelb.edu.au/duet/stability) and INPS-3D (https://inpsmd.biocomp.unibo.it/inpsSuite/default/index3) servers for stability prediction.

#### Molecular docking simulations

To evaluate the impact of different SNPs on ligand interaction, we conducted molecular docking by comparing the wild-type and mutant proteins. The 3D structure of the ligand was obtained from the PubChem database (https://pubchem.ncbi.nlm.nih.gov/) and subsequently optimized using Avogadro software version 1.95.1 (https://avogadro.cc/). Protein-substrate docking was performed using the AutoDock 4.2 program package. During the preparation of the receptor input file, we eliminated water molecules and heteroatoms, and then added missing hydrogens and Gasteiger charges. The conformation space search was conducted within a grid box, with a grid-point spacing of 0.375 Å and dimensions of (40 × 40 × 40) points. Finally, we performed molecular visualization using Discovery Studio 2017 R2 (https://www.3dsbiovia.com/products/collaborative-science/biovia-discoverystudio/).

## Results

### Step1: Collection of Pain management pharmacogenes and variants shared among Mediterranean populations

According to the PRISMA analysis we identified 263 papers investigating SNP involved in pharmacogenomic of pain management through PubMed, Web of Science and Google Scholar databases and an additional 70 papers through pharmGKB database. After removing duplicates, a total of 76 papers were screened for their relevance. Abstracts and titles screening identified 62 studies that met the inclusion criteria. After the full-text analysis, we excluded 20 studies not including genotyping and ethnic data. Hence, 42 studies were included in this study (Figure 2, Supplementary Table S2). Prisma reviewing and PharmGKB database screening have shown that 63 pharmacogenes are involved in the pharmacogenomics of pain management drugs. The results are shown in Table 1, which illustrates the pharmacogene’s name, chromosomal region, related drug, correlated phenotype, context, variant, and therapeutic effect.

**TABLE 1:** Summary of selected pharmacogenes.

Treatment	Pharmacogene	Chromosomic localisation	Phenotype	Context of pain	SNP	Therapeutic effect	Ref
ACETAMINOPHEN	*FAAH*	chr1:46,394,317-46,413,845	Severe cutaneous adverse reactions	Postoperative	rs32442	Toxicity	Planelles et al. (2020)
Aspirine	*CHRNA3*	chr15:78,595,39-78,620,996	Opium deratives	Cancer	rs1051730	Toxicity	Erlich et al. (2010)
	*UGT2B17*	chr4:68,537,176-68,576,322	Coronary Artery Disease; Myocardial Infarction	Postoperative	rs6817882	Toxicity	Kahma et al. (2018)
	*MACROD2*	chr2:13,995,516-16,530,196	Pain	Cancer	rs76026520	Efficacy	Lopes et al. (2022)
	*CYP3A4*	chr7:99,756,967-99,784,184	Pain	Postoperative	rs274574	Efficacy	Chen et al. (2003)
	*UGT2B15*	chr4:68,646,597-68,670,652	Pain	Cancer	rs19223	Efficacy	Pickering et al. (2020)
	*MTRR*	chr5:7,869,148-7,91,113	Aspirin-induced asthma	Postoperative	rs181394	Efficacy	Shin et al. (2014)
	*TGFB1*	chr19:41,33,323-41,353,922	Neoplasms; Pain; Pain, Postoperative	Postoperative	rs18469	Toxicity	Park et al. (2008)
	*STAT6*	chr12:57,95,41-57,111,362	Coronary Artery Disease	Cancer	rs841718	Dosage	Ross et al. (2005)
BUPRENORPHINE	*CYP2C9*	chr1:94,938,658-94,99,91	Opioid-Related Disorders	Postoperative	rs1057910	Efficacy	Wiss et al. (2023)
	*SULT1A1*	chr16:28,65,258-28,623,375	Neonatal Abstinence Syndrome	Cancer	rs1042028	Efficacy	Isvoran et al. (2022)
CODEINE	*CLCC1*	chr1:18,929,56-18,963,484	Pain	Cancer	rs16817	Toxicity	Colombo et al. (2020)
	*OPRD1*	chr1:28,812,17-28,871,267	Pain	Postoperative	rs533123	Dosage	Klepstad et al. (2011)
	*HTR3E*	chr3:184,97,64-184,16,995	Pain	Cancer	rs7627615	Dosage	Klepstad et al. (2011)
	*HTR3D*	chr3:184,32,831-184,39,369	Pain	Postoperative	rs6792482	Dosage	Klepstad et al. (2011)
CODEINE, OXYCODONE	*CYP2D6*	Chr22:42,126,499-42,13,81	Pain	Cancer	rs35742686	Efficacy	Bugada et al. (2020)
DESFLURANE; ENFLURANE; HALOTHANE; ISOFLURANE; METHOXYFLURANE SEVOFLURANE; SUCCINYLCHOLIN	*ZNF568*	chr19:36,916,332-36,952,734	Malignant Hyperthermia	Postoperative	rs145238	Toxicity	Colombo et al. (2020)
	*OPRM1*	Chr6:154,86,57-154,132,356	Malignant Hyperthermia	Postoperative	rs1799971	Efficacy	Klepstad et al. (2011)
	*CYP2C19*	chr1:94,762,681-94,855,547	Malignant Hyperthermia	Postoperative	rs4986893	Efficacy	Wang et al. (2016)
	*ABCC3*	chr17:5,634,881-5,692,253	Malignant Hyperthermia	Cancer	rs4793665	Efficacy	Venkatasubramanian et al. (2014)
	*OPRK1*	chr8:53,225,724-53,251,637	Malignant Hyperthermia	Cancer	rs1051660	Efficacy	Chatti et al. (2017)
	*ARRB2*	chr17:4,710,632-4,721,497	Malignant Hyperthermia	Cancer	rs1045280	Efficacy	Muriel et al. (2019)
	*HTR2C*	chrX:114,584,78-114,91,61	Malignant Hyperthermia	Neuropathic	*rs6318*	Efficacy	González-Castro et al. (2017)
	*HINT1*	chr5:131,159,283-131,165,348	Malignant Hyperthermia	Postoperative	rs255138	Dosage	Klepstad et al. (2004)
	*DRD2*	chr11:113,49,65-113,475,398	Malignant Hyperthermia	Cancer	rs6275	Dosage	Cargnin et al. (2014)
	*TNF*	chr6:31,575,565-31,578,335	Malignant Hyperthermia	Postoperative	rs18629	Dosage	Klepstad et al. (2011)
	*HTR3B*	chr11:113,98,841-113,946,561	Malignant Hyperthermia	Cancer	rs11214763	Dosage	Stamer et al. (2013)
	*ADRA2A*	chr1:111,77,29-111,8,94	Malignant Hyperthermia	Cancer	rs553668	Dosage	Kurita et al. (2016)
	*HTR3A*	chr11:113,975,18-113,99,312	Malignant Hyperthermia	Cancer	rs1176719	Dosage	Klepstad et al. (2011)
	*CNR1*	chr6:88,139,866-88,166,347	Malignant Hyperthermia	Cancer	rs12720071	Dosage	Klepstad et al. (2011)
	*TACR1*	chr2:75,46,463-75,199,52	Malignant Hyperthermia	Postoperative	rs12713837	Dosage	Bright et al. (2021)
	*GCH1*	chr14:54,842,17-54,92,826	Malignant Hyperthermia	Postoperative	rs752688	Dosage	Klepstad et al. (2011)
	*UGT2B7*	Chr4:69,96,474-69,112,987	Malignant Hyperthermia	Postoperative	rs7439366	Dosage	Blanco et al. (2016)
FENTANYL	*COMT*	Chr22:19,941,772-19,969,975	Pain		rs4680	Toxicity	Saiz-Rodríguez et al. (2019)
	*CYP2B6*	chr19:4,991,282-41,18,398	Pain	Postoperative	rs3745274	Toxicity	Bright et al. (2021)
	*CHRNA5*	chr15:78,565,52-78,595,269	Pain	Postoperative	rs16969968	Toxicity	Erlich et al. (2010)
	*TRPV1*	chr17:3,565,446-3,592,877	Pain	Postoperative	rs224534	Efficacy	Pickering et al. (2020)
	*CYP1B1*	chr2:38,67,51-38,76,151	Pain postoperative	Postoperative	rs1056837	Efficacy	Lopes et al. (2022)
	*SLC6A4*	chr17:3,194,319-3,235,697	Pain	Cancer	rs1042173	Efficacy	Zhu et al. (2020)
	*HTR2A*	chr13:46,831,546-46,897,53	Pain	Cancer	rs6313	Efficacy	Cargnin et al. (2014)
	*MTHFR*	chr1:11,785,73-11,83,677	Pain, Postoperative	Neuropathic	rs1801133	Efficacy	Menon et al. (2012)
	*HRH1*	chr3:11,137,238-11,263,557	Pain	Postoperative	rs2606731	Dosage	Klepstad et al. (2011)
MORPHINE	*KCNJ6*	chr21:37,67,373-37,916,457	Pain	Postoperative	rs6517442	Efficacy	Elens et al. (2016)
	*CYP3A5*	chr7:99,667,824-99,679,996	Neoplasms; Pain; Pain, Postoperative	Postoperative	rs776746	Toxicity	Elens et al. (2016)
	*POM121L2*	chr6:27,39,63-27,312,232	Neoplasms; Pain; Pain, Postoperative	Cancer	rs41269255	Toxicity	Colombo et al. (2020)
	*NFKB1*	chr4:12,51,359-12,617,32	Neoplasms; Pain; Pain, Postoperative	Postoperative	rs230493	Toxicity	Liu et al. (2018)
	*ABCB1*	chr7:87,53,17-87,713,295	Neoplasms; Pain; Pain, Postoperative	Cancer	rs1045642	Efficacy	Benavides et al. (2020)
	*TRPA1*	chr8:72,21,25-72,75,584	Anemia, Sickle Cell	Cancer	rs92829	Efficacy	Jhun et al. (2018)
	*TAOK3*	Chr12:118,149,81-118,372,97	Pain Postoperative	Postoperative	rs795484	Dosage, Efficacy	Cook-Sather et al. (2014)
	*SLC22A1*	chr6:16,121,815-16,158,718	Postoperative Pain	Postoperative	rs12208357, rs34130495	Dosage	Tzvetkov et al. (2011)
O-DESMETHYLTRAMADOL; TRAMADOL	*SLC9A9*	chr3:143,265,222-143,848,468	Pain	Postoperative	rs483963	Efficacy	Nishizawa et al. (2018)
	*HTR3C*	chr3:184,53,47-184,6,673	Pain	Postoperative	rs676641	Dosage	Klepstad et al. (2011)
	*HTR1A*	chr5:63,957,876-63,962,445	Pain	Postoperative	rs1423691	Dosage	Klepstad et al. (2011)
	*HTR4*	chr5:148,481,552-148,654,527	Pain	Cancer	rs1971431	Dosage	Klepstad et al. (2011)
OPOIDS	*GNAZ*	chr22:23,7,519-23,125,32	Pain	Cancer	rs3788339	Dosage	Klepstad et al. (2011)
OXYCODONE	*MUC16*	chr19:8,848,844-9,1,437	Pain	Cancer	rs11882256	Toxicity	Colombo et al. (2020)
	*CRYBG2*	chr1:26,321,859-26,354,13	Pain	Postoperative	rs36024412	Toxicity	Colombo et al. (2020)
	*BLMH*	chr17:3,248,23-3,291,944	Pain	Cancer	rs1050565	Toxicity	Lavanderos et al. (2019)
	*ATM*	chr11:18,223,67-18,369,12	Pain	Postoperative	rs1121257	Toxicity	Liu et al. (2018)
	*RHBDF2*	chr17:76,47,896-76,51,423	Pain	Postoperative	rs12948783	Efficacy	Galvan et al. (2011)
TRAMADOL	*PDE3A*	chr12:2,368,537-2,688,583	Pain	Cancer	rs123538	Toxicity	Colombo et al. (2020)
	*IL1A*	chr2:112,773,926-112,784,4	Pain	Cancer	rs18587	Efficacy	McClay et al. (2011)
TRAMADOL, OXYCODONE, MORPHINE	*COMT*	Chr22:19,941,772-19,969,975	Pain	Cancer	rs4633	Efficacy	Lavanderos et al. (2019)

Italic values indicate gene names.

### Step 2: Genetic landscape of pain management variants

#### Statistical analysis

**FIGURE 3:**
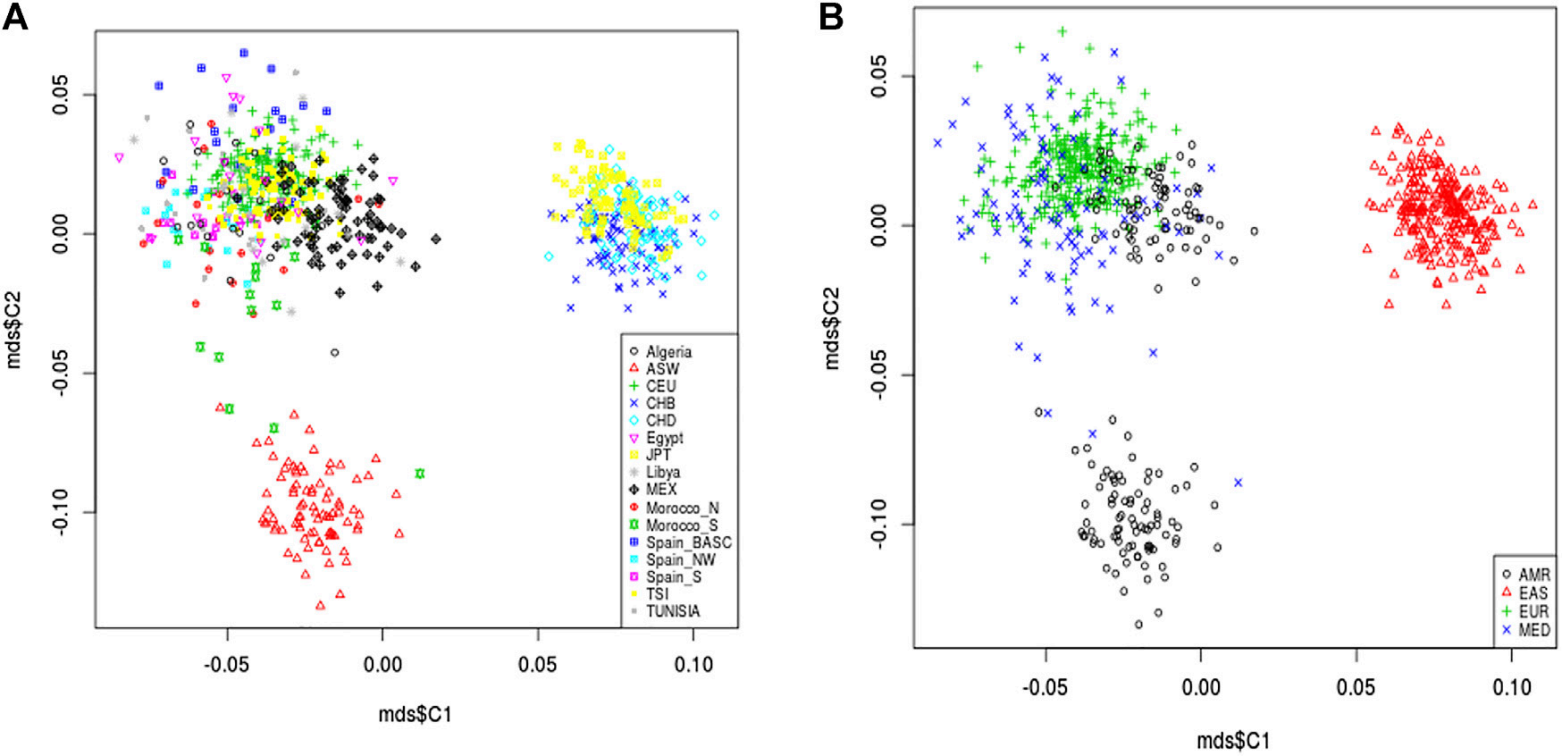
Multidimensional scaling plot of pain management pharmacogene shared variants. The plot shows the proximity of North African populations (Algeria, Egypt, Libya, Morocco-N, Morocco-S, Tunisia) and the European populations (CEU, Sapin-S, Spain-Basic, Spain-NW, and TSI) in the Mediterranean region which are clustered together and distinguished from the American (ASW, MEX) and Asian (CHB, CHD, JPT) populations **(A)**. Better individualization was observed in MDS performed across continents. In addition, there is a great genetic divergence among the Mediterranean (MED), American (AMR) and East Asian (EAS) groups. However, slight proximity was observed between the MED cluster and AMR from Mexico **(B)**.

Variants located in pain management 63 pharmacogenes were extracted from the genotyping data of 829 studied individuals. A total of 1,450 common (p.value >5 × 10^−2^) and rare variants (p.value <5 × 10^−2^) were retained after quality control and genotypic data pruning. MDS analysis describing the genetic landscape of these genetic variants showed that North African populations (Algeria, Egypt, Libya, Morocco-N, Morocco-S, Tunisia) were clustered within the European populations (CEU, Spain-S, Spain-Basic, Spain-NW, and TSI) and have slight proximity to American population from Mexico. We showed that North African and European populations were distinguished from the American (ASW) and Asian (CHB, CHD, JPT) populations (Figure 3). Better individualization was observed in the MDS performed across continents. We observed a great genetic divergence between the Mediterranean (MED), American (AMR), and East Asian (EAS) groups. However, slight proximity was found between the MED and MEX populations.

#### Inferring admixture events between Mediterranean populations

**FIGURE 4:**
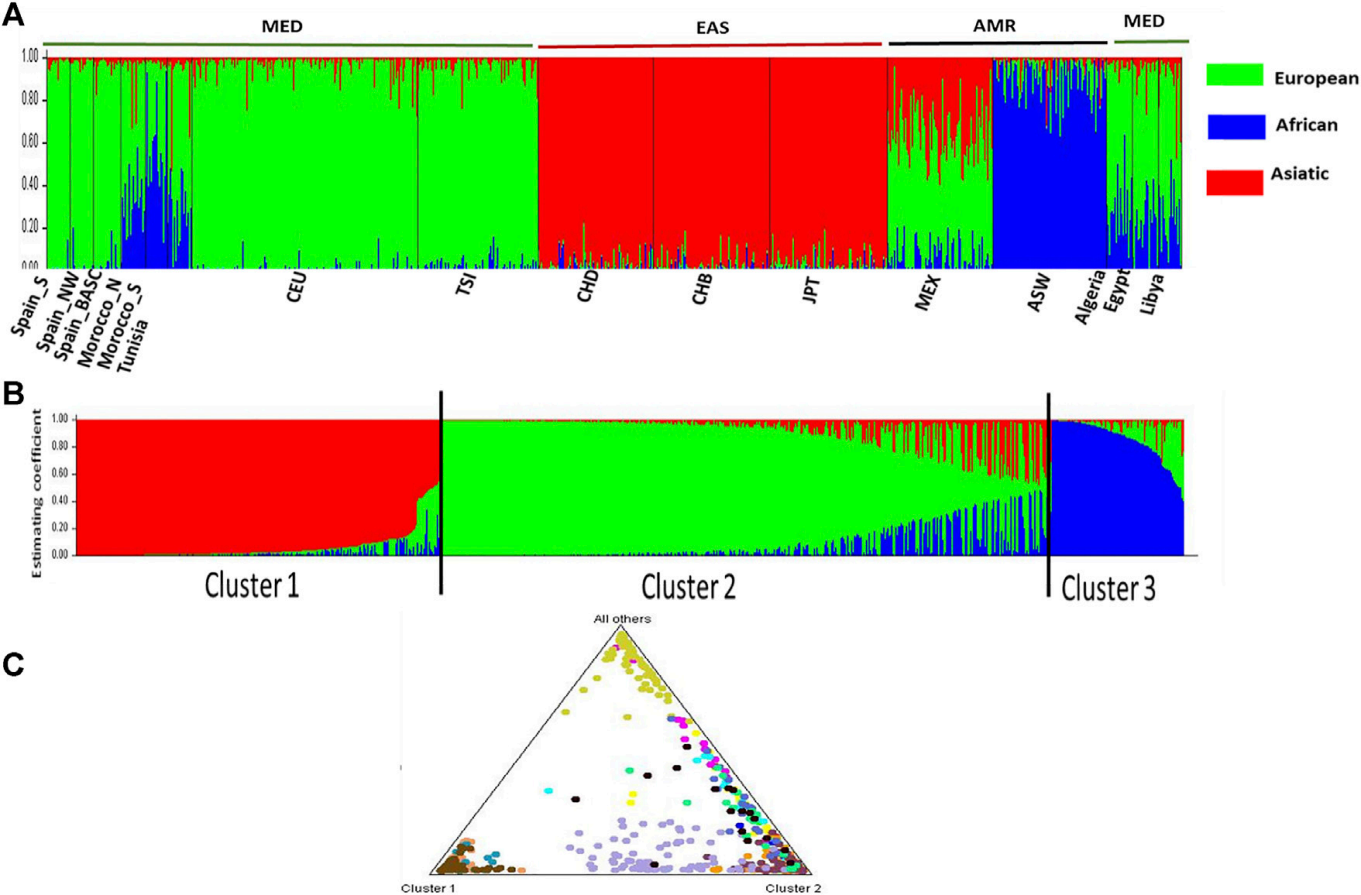
STRUCTURE analysis Bar plot (K = 3). **(A)** describes the genetic relationship between 16 populations at the best estimated k level. K presents the possible numbers of parental population clusters. One color represents one parental population into different color segments. The proportion of each ancestral component in a single individual is represented by a vertical bar divided into 3 colors red, green and blue. In this analysis, 1,450 markers study display results for runs with highest likelihood out of 16 runs in each cluster K3 to 16. Black vertical lines identify the population boundaries. The height extent of each color within an individual’s color bar corresponds to the estimated membership of the individual in one of the clusters; each cluster is assigned a separate color. The bars with multiple colors can be interpreted as genetic admixture or as relative probabilities of belonging to the different clusters. **(B)** Three estimated clusters of the 829 individuals presented with different colors inferred by STRUCTURE analysis. Clusters 1–3 were presented by red, green, blue, and yellow, respectively. Each bar represents an individual, in which a different color represents the estimated membership coefficients using the Q statistic. **(C)** The triangle result shows that the North African populations are close to the European, forming a cluster 2 which is distinct from the EAS cluster and AMR cluster.

The inference of admixture events to the overall pharmacogene variation in pain management was estimated using STRUCTURE software. Ancestry proportions for the studied individuals were estimated from hypothetical ancestors K = 2 to K = 16. The best K value estimated using the Evanno’s ΔK method was 3 (Supplementary Figures S1, S2). We observed the emergence of specific population clusters at this ancestry (K = 3). The ancestry assignment was mainly differentiated between African (blue), European (green), and Asian (red). The Mediterranean population (MED) was clearly differentiated from the American (AMR) and Asiatic populations (EAS) (Figure 4, Supplementary Figure S3). The number of clusters are confirmed by the estimated membership coefficients using the Q statistic (Figure 4B). The Triangle plot shows that the MED are distinct from ASW, EAS and AMR.

#### Allelic frequency of variants with relevant therapeutic effect

Our statistical analysis revealed significant differences in MAF of three variants with high therapeutic effects (rs4633, rs4680, and rs165728) located in the Catechol-O-methyltransferase protein (*COMT*) gene among the MED, AMR, and EAS populations (Table 2). The frequency of the minor allele “T” of rs4633 in the MED populations was higher than the AMR and EAS populations by 36% and 79%, respectively (*p* = 0.0087). On the other hand, the frequency of “A” allele of rs4680 is associated with low frequency in MED compared to AMR and EAS (42% and 57%, respectively) (p.value = 0.019). Moreover, the “C” allele of rs1655728 in the MED population was significantly lower than AMR (*p* = 0.006) and EAS (−75% and −87%, respectively) (*p* = 8.856e-08). The “G” allele of rs13070715 (reindexed within dbSNP as rs2227931) represented higher frequencies in the MED than AMR and EAS studied populations (43% and 12%, respectively).

**TABLE 2:** Allelic frequencies comparison of pain therapeutic high impact variants among studied populations.

^Pain Drug^	^Pharmacogene^	^Variant^	^MAG^	^MAF^	^MAF−MED^	^MAG−MED^	^MAF−AMR^	^MAG−AMR^	^p.value^	^IC^	^Diff^ ^(%)(MED−AMR)^	^MAF−EAS^	^MAG−EAS^	^p.value^	^IC^	^Diff^ ^(%)(MED−EAS)^
Morphine	*COMT*	rs4633	C	T	47.65	52.35	35.00	65.00	0.085	0.934–3.150	36	26.63	71.37	**0.0087**	1.212–4.232	79
NSAIDs Ibuprofen	*COMT*	rs6267	G	T	0.31	99.69	5.84	94.15	**0.029**	0.000–0.825	−95	3.831	96.169	0.1212	0.000–1.496	−92
Morphine Oxycodone Propanol	*COMT*	rs4680	G	A	46.17	53.83	32.50	67.50	0.059	0.979–3.356	42	29.41	70.59	**0.01916**	1.117–3.908	57
Morphine	*COMT*	rs165728	T	C	4.55	95.46	18.44	81.50	**0.007**	0.067–0.713	−75	35.29	64.71	**8.86E-08**	0.028–0.271	−87
Opioids	*ATR*	rs130707	A	G	40.71	59.29	28.44	71.56	0.073	0.950–3.376	43	36.27	63.73	0.561	0.671–2.275	12

MAF, minor allele frequency; MAG, major allele frequency; MED_MAF, derived allele frequency in the Mediterranean population; AMR_MAF, derived allele frequency in the American population; EAS_MAF, derived allele frequency in the East Asiatic population, p. value are corrected with Bonferroni test. P. value is divided on number of variant. P. value threshold is 0.01. Diff % is the minor allele frequency differences among MED, populations compared to EAS and AMR, populations, expressed in percentage.

Bold values indicate variant with significant differences (*p* value < 0.01).

### Step 3: Variant functional analysis

#### Pathway enrichment analysis

**FIGURE 5:**
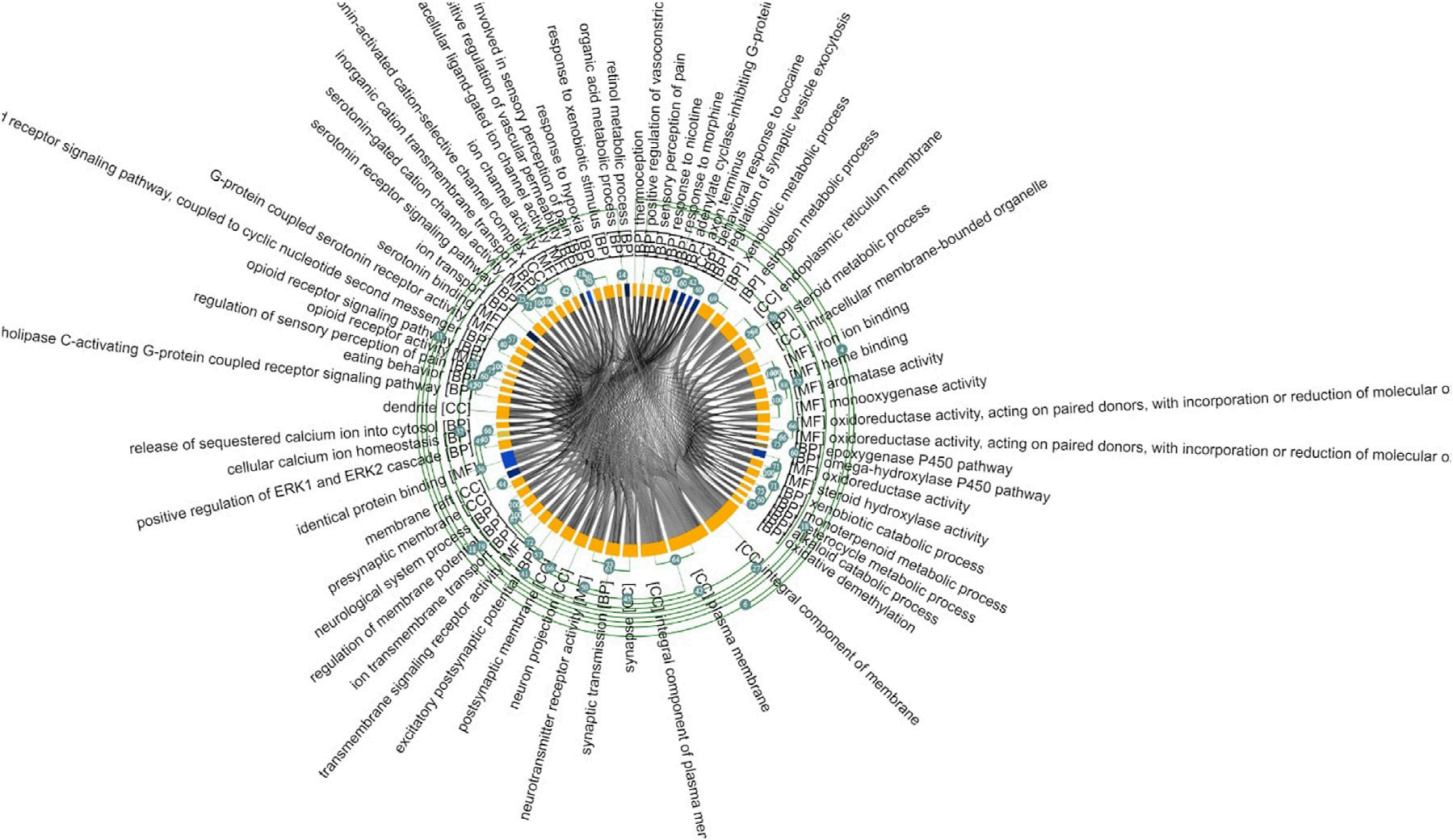
MonaGO chord diagram of enriched pathways related to pain management pharmacogenes

We performed a pathway enrichment analysis of the obtained gene list using the DAVID online tool. A total of 47 biological pathways were enriched in pain management genes with *p*-value <0.05. Results are shown in Table 3. The MonaGO chord diagram showed that pain management genes were enriched in 30 GO biological functions with cut-off *p*-value<2 × 10^−4^ (Figure 5). The most GO enriched terms were: xenobiotic metabolic process, serotonin receptor signaling pathway, estrogen metabolic process, excitatory postsynaptic potential, and steroid metabolic process. The most enriched KEGG pathways were: Serotonergic synapse, Drug metabolism—cytochrome P450, Chemical carcinogenesis—DNA adducts, and Neuroactive ligand-receptor interaction. Wikipathway revealed four significant pathways enriched in pain management: Steroid metabolism, Behavior, Ion transport, and Lipid metabolism. Whereas BIOCARTA showed one significant pathway; Nuclear Receptors in Lipid Metabolism and Toxicity (*p*-value = 2.09 × 10^−4^) (Supplementary Table S3).

**TABLE 3:** Pathway enrechiment analysis result.

GO	Go-term	*p*-value	Number of genes
GO:0006805	xenobiotic metabolic process	6.023204611239894E-16	13
GO:0007210	serotonin receptor signaling pathway	4.308385330705686E-15	7
GO:0008210	estrogen metabolic process	3.3945426412321174E-14	9
GO:0060079	excitatory postsynaptic potential	1.3116645492262196E-13	11
GO:0008202	steroid metabolic process	3.6226813879482314E-12	9
GO:0007187	G-protein coupled receptor signaling pathway, coupled to cyclic nucleotide second messenger	1.026584159142599E-9	7
hsa00982	Drug metabolism - cytochrome P450	2.0496611403446925E-11	11
hsa05204	Chemical carcinogenesis - DNA adducts	1.0826076922649092E-8	9
KW-0085	Behavior	4.615367305097824E-6	4
KW-0406	Ion transport	5.421628769811925E-6	13
KW-0443	Lipid metabolism	1.8399442428564817E-5	13
h_nuclearRsPathway	Nuclear Receptors in Lipid Metabolism and Toxicity	2.094043157401423E-4	5

#### MiRNAs analysis

**FIGURE 6:**
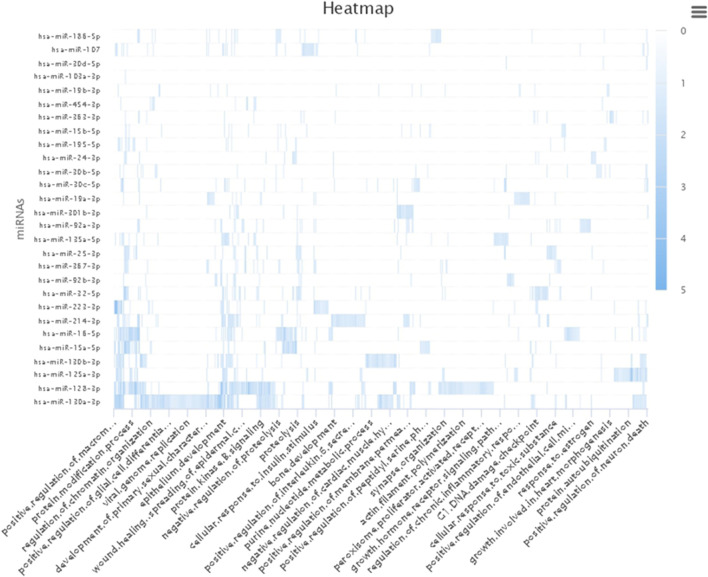
Heatmap of pain pharmacogene miRNAs enriched pathways. Our result showed that miRNAs were mapped to several pathways involved in regulation of acute inflammatory response, cellular calcium ion homeostasis, response to drug, cellular response to DNA damage, cellular response to toxic substance, regulation of chronic inflammation response, synaptic transmission GABAergic, response to estrogen, and positive regulation of metabolic process.

The SNPinfo shows that ten variants have potential miRNA-binding sites (Table 4). After the investigation of miRdSNP database, we noticed that only eight out of ten variants dispose of potential miRNA-binding sites. Pathway analysis revealed that detected miRNA was enriched in multiple GO pathways. A number of these pathways was also identified by our GO pathway analysis of pain management associated genes (Figure 6). These pathways are: regulation of acute inflammatory response, cellular calcium ion homeostasis, response to drug, cellular response to DNA damage, cellular response to toxic substance, regulation of chronic inflammation response, synaptic transmission GABAergic, response to estrogen, and positive regulation of metabolic process. The main enriched miRNAs associated with these pathways were: hsa-miR-130a-3p, hsa-miR-223-3p, hsa-miR-15a-5p, hsa-miR-128-3p, hsa-miR-30c-5p, hsa-miR-19a-3p, andhsa-miR-24-3p. Results are shown in Table 4.

**TABLE 4:** Summary of variants with potential effect on miRNAs binding sites.

Position	Mapped gene	SNP	miRNA
1:203166324	*ADORA1*	rs16851030	hsa-miR-24
2:138014190	*HNMT*	rs1050891	hsa-miR-186, hsa-miR-873, hsa-miR-223
2:233772770	*UGT1A10*	rs10929303	hsa-miR-371-5p
2:233772898	*UGT1A10*	rs1042640	hsa-miR-371-5p
2:233772999	*UGT1A10*	rs8330	hsa-miR-371-5p
8:132010916	*EFR3A*	rs4736529	hsa-miR-92a, hsa-miR-32, hsa-miR-367, hsa-miR-363, hsa-miR-92b, hsa-miR-25, hsa-miR-30b, hsa-miR-30e, hsa-miR-30c, hsa-miR-30a, hsa-miR-30d, hsa-miR-125a-3p, hsa-miR-128, hsa-miR-19b, hsa-miR-19a, hsa-miR-301b, hsa-miR-130a, hsa-miR-454
17:30197993	*SLC6A4*	rs1042173	hsa-miR-135a, hsa-miR-135b, hsa-miR-182, hsa-miR-107, hsa-miR-195, hsa-miR-103, hsa-miR-15a, hsa-miR-15b, hsa-miR-16, hsa-miR-214
19:46620526	*PTGIR*	rs1126510	hsa-miR-149, hsa-miR-331
1:203166324	*ADORA1*	rs16851030	hsa-miR-24
2:138014190	*HNMT*	rs1050891	hsa-miR-186, hsa-miR-873, hsa-miR-223
2:233772770	*UGT1A10*	rs10929303	hsa-miR-371-5p
2:233772898	*UGT1A10*	rs1042640	hsa-miR-371-5p
2:233772999	*UGT1A10*	rs8330	hsa-miR-371-5p
8:132010916	*EFR3A*	rs4736529	hsa-miR-92a, hsa-miR-32, hsa-miR-367, hsa-miR-363, hsa-miR-92b, hsa-miR-25, hsa-miR-30b, hsa-miR-30e, hsa-miR-30c, hsa-miR-30a, hsa-miR-30d, hsa-miR-125a-3p, hsa-miR-128, hsa-miR-19b, hsa-miR-19a, hsa-miR-301b, hsa-miR-130a, hsa-miR-454
17:30197993	*SLC6A4*	rs1042173	hsa-miR-135a, hsa-miR-135b, hsa-miR-182, hsa-miR-107, hsa-miR-195, hsa-miR-103, hsa-miR-15a, hsa-miR-15b, hsa-miR-16, hsa-miR-214
19:46620526	*PTGIR*	rs1126510	hsa-miR-149, hsa-miR-331

Italic values indicate gene names.

#### Predicting the structure impact of nsSNP

**FIGURE 7:**
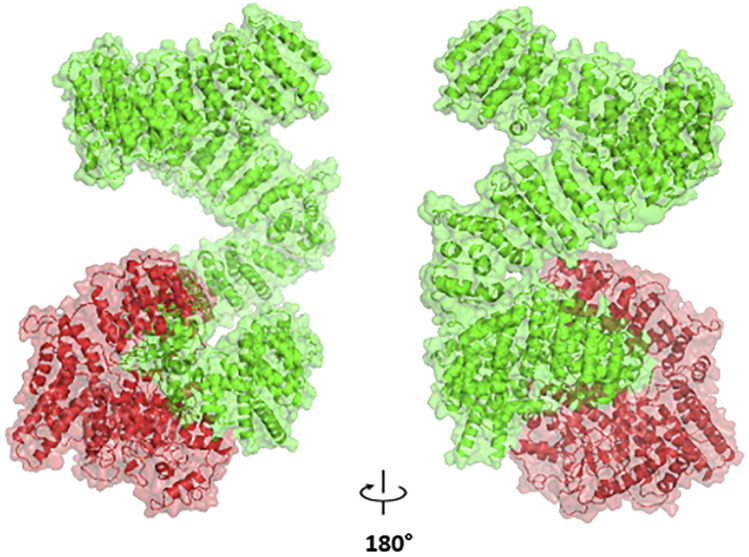
The Impact of p. Tyr1736Ter Nonsense Variant (rs2227931) on the ATR Protein: depiction of the truncated protein segment highlighted in red.

We focused our structural analysis primarily on three non-synonymous single nucleotide polymorphisms “nsSNPs” (rs4680, rs6267, and rs2227931). The first two variants (rs4680, and rs6267) are located in the COMT gene. We utilized the crystal structure of COMT (PDB ID: 3IBW) with a resolution of 1.93 Å to create the corresponding mutated proteins. The RMSD analysis revealed slight deviations of 0.179 Å and 0.187 Å, respectively. Moreover, the tools: mCSM, SDM, DUET and INPS-3D predicted that these mutations would destabilize the protein, as indicated by negative ΔΔG values of −0.697, −1.250, −0.857 and −0.381 kcal/mol for rs6267, and -1.372, −2.450, −1.507 and −0.74 kcal/mol for rs4680. The rs2227931 SNP is located within the ATR serine/threonine kinase ATR gene and causes a premature stop codon at position 1736. As a consequence, this mutation leads to the loss of 34% (908 amino acids) of the protein sequence (Figure 7). Specifically, it results in the deletion of the Kinase domain, PIKK regulatory domain (PRD), FAT C-terminus domain (FATC), and a significant portion of the FAT domain. These alterations in the protein structure may disrupt the function of the ATR protein.

#### Molecular docking and binding energy calculation

**FIGURE 8:**
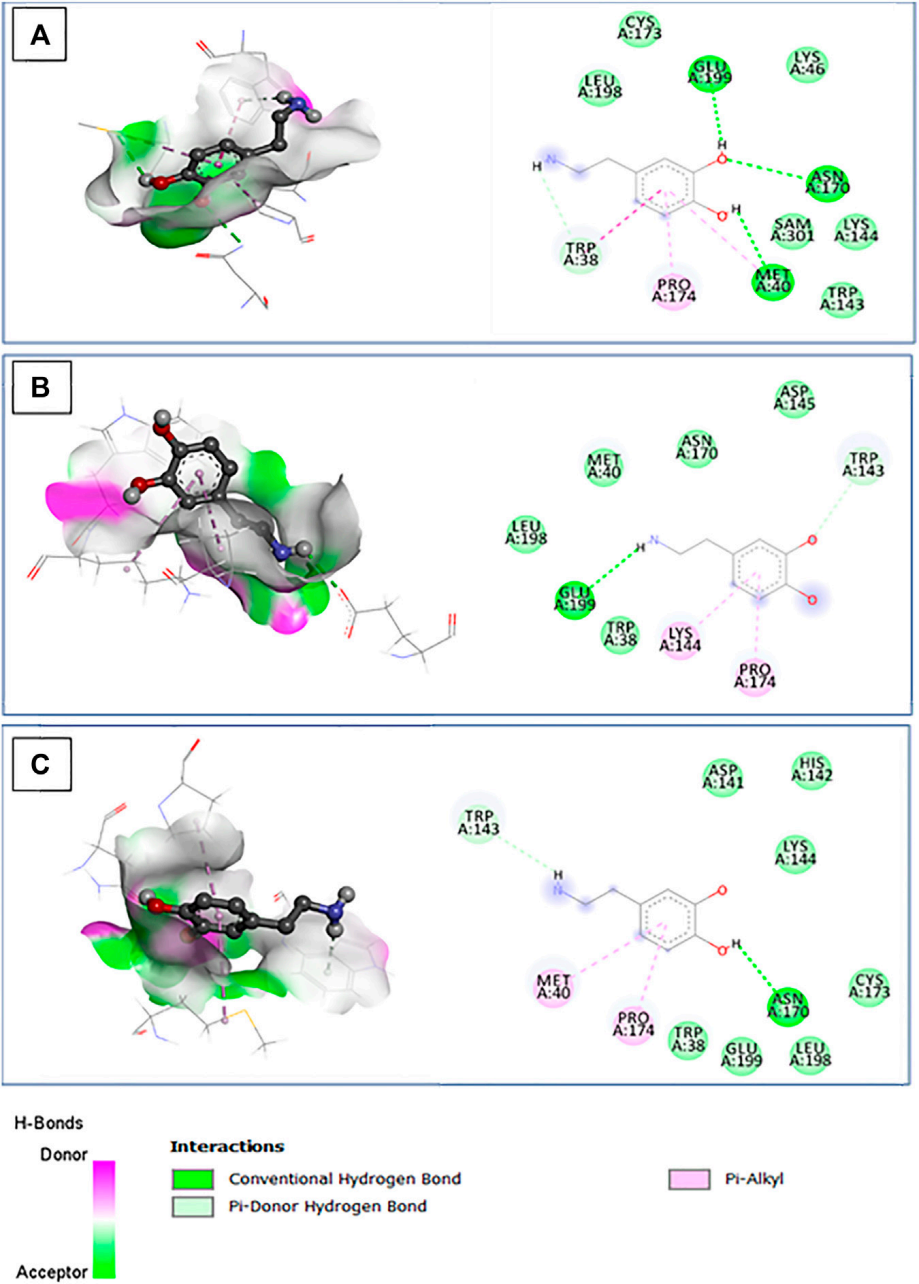
The 3D structure of the docked dopamine bound to the pocket region of the wild type **(A)** and mutant COMT protein **(B)**: rs4680; **(C)**: rs6267, along with hydrogen bonds and the corresponding 2D diagram of the interactions.

In our study, we employed a molecular docking approach to investigate the interaction between wild-type and mutant COMT proteins, specifically rs4680 and rs6267 variants, with dopamine. The calculation of binding energy plays a pivotal role in discerning the affinity between the protein and ligand. By conducting a thorough analysis of the docking complex, we were able to identify noteworthy characteristics. Notably, we observed a decrease in binding energy for the dopamine complex in comparison to the wild-type protein complex (Table 5). This decrease suggests a decline in stability, which may potentially lead to lower enzyme activity. Furthermore, we noticed a decrease in the number of interactions in these complexes (Figure 8). In the wild-type protein complex, three hydrogen bonds were observed with Met40, Asn170, and Glu199, which are specific substrate binding residues. However, in the mutant proteins rs4680 and rs6267, only one hydrogen bond was present with Glu199 and Asn170, respectively.

**TABLE 5:** Dopamine interactions with the active site of COMT wild type and mutant protein: binding affinity, number of conventional hydrogen bonds, and interacting amino acid residues.

Ligand	SNP ID	Residue	Binding affinity (kcal/mol)	Intermolecular interactions	
				Conventional hydrogen bonds	Interacting amino acid residues
Dopamine	Wild type	Val158	−4.9	3	Trp38, Met40^a^, Asn170^a^, Pro174, Glu199^a^
		Ala72			
	rs4680	Met158	−4.0	1	174Pro, Lys144, Pro174, Glu199^a^
	rs6267	Thr72	−4,1	1	Met40, Asn170^a^, Pro174

^a^ Hydrogen bond.

## Discussion

Differences in allele frequencies may introduce genetic diversity among different ethnic groups which has the potential to alter the therapeutic efficacy of commonly used treatments (Goodman and Brett, 2021) such as analgesics. Therefore, incorporation of pharmacogenomic assessments prior to treatment would be extremely beneficial in terms of cost, quality of life, and therapeutic resource optimization.

Indeed, pharmacogenetics relies on genotype-based prescribing decisions to maximize efficacy and mitigate side effect risks. Due to the limited access to genotyping tests, ancestral markers are often used to estimate the likely genotype of a patient. This is based on the frequency of genetic variation in a patient’s ethnic group, as some variations are specific to certain ethnic groups. Thus, a deeper understanding of ancestral pharmacogenetic markers may lead to a better understanding of pharmacogenetic mechanisms in Mediterranean populations, which could enhance the development of tailored pain therapies for these populations (Shah and Gaedigk, 2018). Moreover, the ancestral marker data may be very helpful in developing pharmacogenetic tests that can be easily translated into clinical care practices. As a result, it can reduce the socio-economic and psychological burdens of pain conditions. Furthermore, reducing the harmful toxicities and risk of addiction associated with pain care requires the discovery of these ancestral markers. Hence, a wide representation of different ethnic groups is essential for the development of tailored pain management strategies. Pharmacogenetics studies are mainly conducted on white-non hispanic population (Ortega and Meyers, 2014), and little is still known about the pharmacogenetics of the Mediterranean basin, especially the southern countries of this region. In the present study, we investigated the pharmacogenetic landscape of variants implicated in pain management across the Mediterranean region, compared to other groups of populations. Next, we explored their role in different biological processes to identify potential therapeutic targets. Finally, we studied the protein-drug reaction to better understand the role of these variants in the therapeutic efficacy and toxicity.

### Genetic landscape of pain management

Our MDS analysis revealed a high genetic similarity between North African and European populations of the MED region. However, we observed a great genetic divergence between the MED populations group and the two other groups (AMR and EAS). These results were further validated when conducting admixture analysis. Indeed, we noticed that the MED group is composed mainly of European and African components with low genetic contribution of the EAS ancestries. These findings are consistent with previous results demonstrating the impact of ethnicity on genetic variability of pain pharmacogenes (Perry et al., 2019). In fact, a similar genetic positioning was also observed in a previous study conducted on pharmacogenes implicated in metabolic syndrome drug response (Jmel et al., 2018). Furthermore, Mezzi et al., demonstrated a high genetic homogeneity between European populations (Mizzi et al., 2014). These results could be explained by the ancestral invasion and migration events especially between the North African populations and the South-west European populations. On the other hand, the high genetic heterogeneity between MED group and EAS populations could be due to an ancient divergence between the two groups (Boukhalfa et al., 2023). Interestingly, we notice a genetic proximity between MED populations and Mexican populations. This could be explained by the colonial history of Mexico, especially from circum-Mediterranean regions of Spain (Gómez et al., 2021).

The high genetic admixture of the MED populations in comparison to other ethnic groups could explain their high genetic variability regarding pain management pharmacogenes. Indeed, the minor allele frequency (MAF) comparison has shown the difference in MAF of three variants (rs4680, rs4633, and rs165728) located in COMT gene. The COMT gene encodes the catechol-O-methyltransferase, a key enzyme that regulates cognitive functions, mood and pain perception through regulating the catecholamine concentrations (Sadhasivam et al., 2014). The first variant, rs4680 (G/A) induces an amino acid change from Valine to Methionine. It was predicted to induce COMT protein instability. Our docking analysis revealed a decrease in binding energy and a reduction in the number of interactions. These findings align with previous studies in the literature, which have indicated that the presence of the A allele is associated with lower enzymatic activity, consequently leading to elevated levels of prefrontal dopamine (Mir et al., 2018). Our results showed a lower frequency of the “A” allele of rs4680 in MED compared to AMR and EAS population. This variant is associated with increased analgesic response to morphine (Elens et al., 2016). According to a study conducted by Rakvåg et al., Indian individuals with cancer from various sources (breast, lung, abdominal cavity, and urogenital system) who had the GG genotype of the *COMT* rs4680 received an average of 50% higher daily doses of morphine compared to those with the AA genotype (Rakvåg et al., 2005). Similarly, a previous study conducted on American patients after nephrectomy showed that carriers of the GG genotype had a higher morphine consumption in comparison to the AA genotype carriers (Candiotti et al., 2014). In contrast, the AA genotype was significantly associated with moderate to higher-pain in South African breast cancer survivors (Firfirey et al., 2022). However, no association between this variant and morphine clinical efficacy in Tunisian cancer patients (Chatti et al., 2017).

In addition, a previous study reported reduced morphine needs related to the rs4680 minor allele (A) in people of European Caucasian and Asian origin. The effects of rs4680 are not consistently supported in pediatricians but only in adults. The rs4680 minor allele A is associated with increased pain during mobilization following surgery and decreased postoperative analgesic administration (Li et al., 2019). In contrast, the minor A-allele of this polymorphism decreases catabolic enzyme activity by 25%, resulting in more dopamine in the prefrontal cortex (Tchivileva et al., 2010). However, the significance of opioids’ stimulating effects to their addiction potential is unknown. Some studies believe that dopamine-mediated reward deficit is an incentive for drug abuse (Blum et al., 2000). This energizing effect might be an endophenotype (Gottesman and Gould, 2003), indicating a desire for non-medical opioid usage. Furthermore, earlier research has linked rs4680 to variations in opioid usage in chronic pain patients after surgery (Yennurajalingam et al., 2021). Previous study has shown that rs4680 decreases COMT enzyme activity, resulting in poor noradrenaline metabolism and increased noradrenaline concentrations in the bloodstream, as well as impacts on the brain’s arousal regions, which explains the decrease in somnolence. The increasing noradrenaline concentration may have also an effect on the analgesic effects (Valomon et al., 2014). A clinical study of cancer pain in Asian patients found that GG carriers require a greater intravenous morphine dosage than AA carriers (Tanaka et al., 2021), with the lack of drug preventing or lowering opioid-induced somnolence (Cargnin et al., 2014). We showed significant MAF differences of rs4633 (C>T) between The MED, AMR and EAS populations. This variant has previously been linked to fibromyalgia in Korean patients (Knezevic et al., 2018b) and postoperative pain in American children (Sadhasivam et al., 2014), where researchers discovered that children with the (CC) genotype require more morphine and analgesic intervention postoperatively than children with the (TT) genotype (De Gregori et al., 2016). The rs4633 variant allele “T” is more common in the MED population than in the AMR and EAS populations. Morphine appeared to be more effective in populations with MED. This finding may influence the morphine dose in MED patients suffering from extreme pain as well as the development of treatment algorithms.

However, allele “C” of rs4633 is less frequent in the MED population than AMR and EAS. This allele is associated with increased severity of Pain when treated with propranolol in Caucasian women with temporomandibular joint disorders and pain (Tchivileva et al., 2010).

The variant rs2227931 located in the overlapping region of the *ATR*-*ATM* genes is highly prevalent in MED and AMR populations than EAS population. This variant is known to be implicated in DNA damage leading to drug resistance. Our modeling result showed that the rs2227931 caused the deletion of the Kinase domain, PIKK regulatory domain (PRD), FATC, and a significant portion of the FAT domain. These alterations in the ATR protein structure disrupt the function of the ATR and lead to opioids resistance (Karran, 2001). Finally, rs165728 is located in the 3′UTR region of the *COMT* gene. A negative association between this variant and opioid daily dose in advanced cancer patients have been recently detected (Yennurajalingam et al., 2021). The study included American patients from three ethnic groups (black non-hispanic, hispanic, and white non-hispanic). Our results pinpointed that the “C” allele of rs165728 is rare in the MED population compared to AMR and EAS studied populations. Moreover, previous studies showed that Allele “C” is associated with increased dose of morphine in Caucasian individuals with Neoplasms as compared to allele T (Rakvåg et al., 2008). We suggest that MED populations require a lower dose of morphine to accomplish the target therapeutic effect.

### Pathway enrichment analysis

We identified 30 enriched GO pathways for pain management. The most enriched GO pathways identified by MonaGO were: xenobiotic metabolic process, serotonin receptor signaling pathway, estrogen metabolic process, excitatory postsynaptic potential, and steroid metabolic process. Xenobiotic metabolic process involves the different pathways implicated in the metabolism of exogenous components like drugs. Serotonin receptor signaling pathway has been widely implicated in stress response as well as in pain signaling (Haleem, 2019). A large body of evidence demonstrates the implication of serotonin1A receptor (5-HT1A receptor) in the control of pain. Thus, multiple therapeutic strategies suggest 5-HT1A receptor as potential pharmacological target for treating pain (Haleem, 2019). Interestingly, there are seven serotonin (5-HT) receptors (5-HT1 to 5-HT7) differentially distributed in the brain (Viguier et al., 2013). A recent study conducted on mild traumatic brain injury (mTBI) showed that 5-HT could enhance the nociception sensitization directly via 5-HT_3_ receptor signaling or indirectly through the upregulation of spinal chemokine production (Sahbaie et al., 2019). Estrogen receptors (ERs) include two nuclear types of receptors (ERα and ERβ), mainly enriched in the cytoplasm and can also be recruited into the cell membrane. Different studies have demonstrated the role of ERs in alleviating nociception (Chen et al., 2021). For example, a previous study has proven the therapeutic effect of Erβ in Inflammatory Bowel Disease (IBD) rat model through the suppression of the P2X purinoceptor 3 receptor (P2X3R) (Jiang et al., 2017). Similarly, Erα poses an anti-nociception action via decreasing ATP-P2X3-mediated Ca2+ influx (Cho and Chaban, 2012). Excitatory postsynaptic potential (EPSE) describes the process that leads to a temporary increase in postsynaptic potential due to the flow of positively charged ions into the postsynaptic cell. A previous study conducted by Ren et al. revealed that monosynaptic EPSPE increased significantly in the arthritis pain rat model through the regulation of metabotropic glutamate receptor subtype 1 (mGluR1) signaling (Ren and Neugebauer, 2010). The steroid metabolic process appears as one of the most enriched pathways in our analysis. Similar results were found regarding therapeutic targets of low back pain (Wen et al., 2022). Steroids and corticosteroids have been widely used as pain relief medications. For example, corticosteroids could directly or indirectly decrease the production of pro-inflammatory cytokines by inhibiting Phospholipase A2 and the ensuing arachidonic acid metabolic pathway (Knezevic et al., 2018a). Genes associated with pain management drugs have been found enriched in multiple other pathways such as: Chemical carcinogenesis - DNA adducts, Neuroactive ligand-receptor interaction, Behavior, Ion transport, and Lipid metabolism. Morphine and related opioids are powerful yet addictive pain drugs. Opium poses several carcinogenic effects. For example, opium smoke could contain carcinogenic chemicals like matic hydrocarbons (PAHs), arsenic and lead. Furthermore, opium alkaloids could induce genotoxic effects such as: chromosome damage, micronuclei, DNA fragmentation related to morphine, and sister chromatid exchanges related to codeine. Finally, opioid receptor activation could enhance angiogenesis and neovascularization, impairment of immune function, and facilitation of tumor initiation, proliferation, and migration (Sheikh et al., 2023). However, contradictory results have been presented regarding their role as chemical carcinogenesis. This discrepancy could be due to the type of the used opium (mixed or pure), condition and intensity (Kosciuczuk et al., 2020; López-Lázaro, 2022). Neuroactive ligand-receptor interaction and Ion transport were significantly enriched in pain management. These findings are in line with those of Chidambaran et al. reporting that Neuroactive ligand-receptor interaction and ion channels were similarly enriched in chronic and acute postoperative pain (Chidambaran et al., 2020). It is widely known that ions, especially calcium (Ca^2+^) plays a critical role in neuronal excitability and synaptic transmission and thus it is inevitable in the pain perception process (Harding and Zamponi, 2022). Interestingly, behavior appears as one of the most enriched pathways in pain management genes. Alterations of brain reward-related behavior have been widely proposed in relation to painkillers use, especially opioids. They can enhance drug-seeking and drug-taking behaviors (Merlin et al., 2018; Grecco and Atwood, 2020; Maumus et al., 2020) specifically through μ opioid receptors (Sikora et al., 2019). Thus, medical professionals should develop a personalized management of patients using opioids. Finally, lipid metabolism plays an important role in chronic pain. For example, a systematic review revealed a significant association between serum HDL, LDL, triglyceride and musculoskeletal pain (Tilley et al., 2015). Furthermore, lipid bioactive mediators such as: endocannabinoids, endogenous PPAR-α activators, and oxidative products of PUFA metabolism appear to regulate the transmission of nociceptive information from peripheral sites of injury and inflammation to the central nervous system (Piomelli et al., 2014).

### MiRNAs analysis

Our *in silico* analysis revealed ten variants were predicted to affect miRNA implicated in several pathways related to pain management such as: regulation of acute inflammatory response, cellular calcium ion homeostasis, response to drug, cellular response to DNA damage, synaptic transmission GABAergic, response to estrogen, and positive regulation of metabolic process. Differential expressions of miR-130a-3p and miR-128-3p have been observed in rat models of spinal cord injury (SCI) (Hu et al., 2021). Recent studies have shown the downregulation of miR-130a-3p in SCI rat models attenuated NP via inhibiting inflammatory markers (IL-1β, IL-6 and TNF-α), mitigated apoptosis, activated microglia and upregulated the IGF-1/IGF-1R signaling axis (Yao et al., 2021). However, that upregulation of miR-128-3p could alleviate the neuropathic pain (NP) in SCI rat models via regulating aquaporin-4 (AQP4) pathway (Xian et al., 2021). Other study suggests the potential role of miR-128-3p in alleviating NP through regulating neuroinflammation mediated by ZEB1 (zinc finger E-box binding homeobox 1) in chronic constriction injury (CCI) rat model (Zhang et al., 2020). ZEB1 can inhibit cell adhesion and has been identified as a potential target of several miRNAs (miR-28-5p, miR-200b, miR429, and miR-150) in neuropathic pain (Zhang et al., 2020). miR-15a is a tumor suppressor in chronic lymphocytic leukemia, pituitary adenomas, and prostate cancer. Recent study by Cai et al. has successfully demonstrated the therapeutic effect of this miRNA in alleviating CCI- induced NP through AKT3 mediated autophagy process (Cai et al., 2020). A previous study suggests that over-expression of miR-30c-5p in plasma and Cerebrospinal Fluid Markers (CSF) in combination with other clinical variables could predict NP in patients with chronic peripheral ischemia (Tramullas et al., 2018). The same study has shown that miR-30c-5p inhibitor delayed neuropathic pain development and reversed fully established allodynia in rodents through regulating mechanisms mediated by TGF-b and involved the endogenous opioid system (Tramullas et al., 2018). Moreover, downregulation of miR-19a-3p appears to mediate NP through MeCP2-mediated BDNF upregulation (Manners et al., 2015). BDNF is a main regulatory factor of sensory neurotransmission in nociceptive pathways and hyperalgesia (Manners et al., 2015). A very recent computational study by Yu et al. (Yu et al., 2023), identified a differential expression of miR-223-3p in NP. These results have been supported by previous findings. Significant downregulation of miR-223-3p has been previously reported in fibromyalgia patients (Bjersing et al., 2013). Furthermore, upregulation of miR-223-3p could alleviate trigeminal neuralgia NP through targeting MKNK2 and MAPK/ERK signals (Huang et al., 2022). In addition, over-expression of miR-223-3p induced by electroacupuncture significantly reduces postherpetic neuralgia pain by inhibiting neuron cell autophagy (Toyama et al., 2017). miR-24-3p is a master regulator of cancer development and occurrence (Wang et al., 2020). Previous study has demonstrated a significant upregulation of miR-24-3p upon opioid treatment suggesting its crucial role in pain management (Toyama et al., 2017). Together, these data demonstrate the potential role of miRNAs in regulating pain sensation through regulation of inflammatory and immune pathways. Finally, these miRNAs could serve as promising biomarkers for monitoring pain-killers activation in the body. Further clinical trials are needed to prove their role in pain management. The implementation of aforementioned biomarkers (SNP and miRNA) in pharmacogenetic testing through genetic and molecular screening of these biomarkers could be advantageous for patient’s tailored treatment (Roberts et al., 2023; Selvaraj et al., 2023). Indeed, genotyping data will be then communicated to the healthcare provider through established Clinical Decision Support Systems (CDSS) or innovative bioinformatic algorithms in order to assess clinical decision-making. This approach will assist physicians in prescribing the most adapted therapeutic molecule and dose for the patient ensuring a personalized and patient-centric approach.

This practices contribute to minimize the side effects and optimize the efficiency of analgesics and opioids. Thus, could be of great interest to better clinical decision-making and reduce socioeconomic disparities such as minimizing healing time and the risk of drug dependency.

## Conclusion

Our findings show that Mediterranean populations are characterized by high genetic heterogeneity in terms of the investigated pharmacogenes involved in chronic pain management. Our results highlight the important role of the identification of ancestral markers, miRNAs and pain related pathways for better clinical decision making and pain management. We suggest that the implementation of these markers through effective pharmacogenetic strategies could improve pain response rate, reduce side effects and healing-time. However, further research is needed to close this gap and explore pain-related pharmacogenetic markers in under-represented population such as Mediterranean populations.

## Data Availability

Publicly available datasets were analyzed in this study. This data can be found here: www.internationalgenome.org, https://journals.plos.org/plosgenetics/article?id=10.1371/journal.pgen.1002397, https://www.science.org/doi/10.1126/science.1153717?url_ver&equals;Z39.88-2003&rfr_id&equals;ori:rid:crossref.org&rfr_dat&equals;cr_pub%20%200pubmed. Generated results are available in the Supplementary Material of the paper.

## Abbreviations

IASP, International Association for the Study of Pain; ADR, Adverse Drug Reactions; COMT, Catéchol-O-Méthyl-Transférase; OPRM1, Opioid Receptor Mu 1; PharmGKB, Pharmacogenomics Knowledge Base; VIP, Very Important Pharmacogene; miRNA, microRNA; HWE, Hardy-Weinberg equilibrium; MDS, Multidimensional scaling plot; IBS, Identity-by-state; PEA, Pathway Enrichment Analysis; GO, Gene Ontology; KEGG, Kyoto Encyclopedia of Genes and Genomes; UP_KW, WikiPathway; BBID, BioCarta Pathways and the Biological Biochemical Image Database; MonaGO, Monash Gene Ontology; RMSD, Root Mean Square Deviation; SNP, Single Nucleotide Polymorphisms; MAF, Minor Allele Frequency; MED, Mediterranean; AMR, American; EAS, East Asiatic; ERs, Estrogen receptors; IBD, Inflammatory Bowel Disease; EPSE, Excitatory postsynaptic potential; mGluR1, Regulation of Metabotropic Glutamate Receptor subtype 1; HDL, Height, Density Lipoprotein; LDL, Low Density Lipoprotein; SCI, Spinal Cord Injury (SCI); FATC, FAT C-terminus domain; CSF, Cerebrospinal Fluid Markers.
