# Supplementary material for: Pharmacogenetic landscape of pain management variants among Mediterranean populations
Source: Front Pharmacol. 2024 May 15;15:1380613. doi: 10.3389/fphar.2024.1380613 (PMC11134176; doi:10.3389/fphar.2024.1380613)
Supplement: Supplementary file 1 [file DataSheet1.PDF]

Supplementary Material

Pharmacogenetic landscape of pain management variants among Mediterranean populations

Authors: Haifa JMEL [1,2,3] Wided BOUKHALFA [1,2,3,4], Ismail GOUIZA[1,2,3,4,5], [1]Roua OULED SEGHAIER, Hamza DALLALI[1,2,3]and Rym KEFI[1,2,3*]

[1] Laboratory of Biomedical Genomics and Oncogenetics, Institut Pasteur de Tunis, Tunis, Tunisia

[2] Genetic typing service, Institut Pasteur de Tunis, BP 74, 13 Place Pasteur, Tunis 1002, Tunisia

3 Tunis El Manar University, Tunis, Tunisia,

4 Faculty of Medicine of Tunis, Tunis, Tunisia

5 University of Angers, MitoLab Team, Unité MitoVasc, UMR CNRS 6015, INSERM U1083, SFR ICAT, Angers, France

*** Correspondence:** Corresponding Author: *rym.kefi@pasteur.utm.tn

1 Supplementary Figures and Tables

1.1 Supplementary Figures

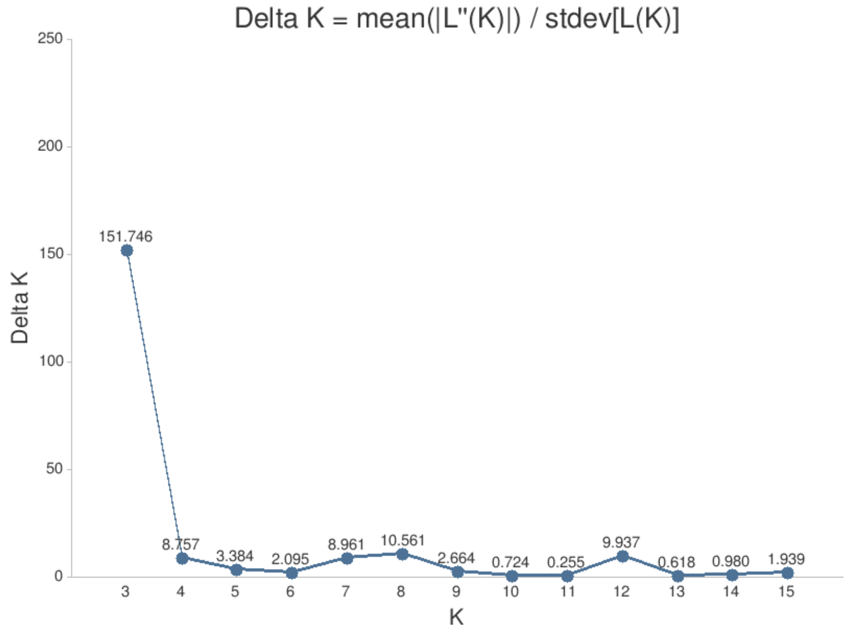

Supplementary Figure 1. Best_K_By_Evanno-Delta K By K graph.

The graph shows the best K equal to 3 according to delta K as proposed by Evanno.

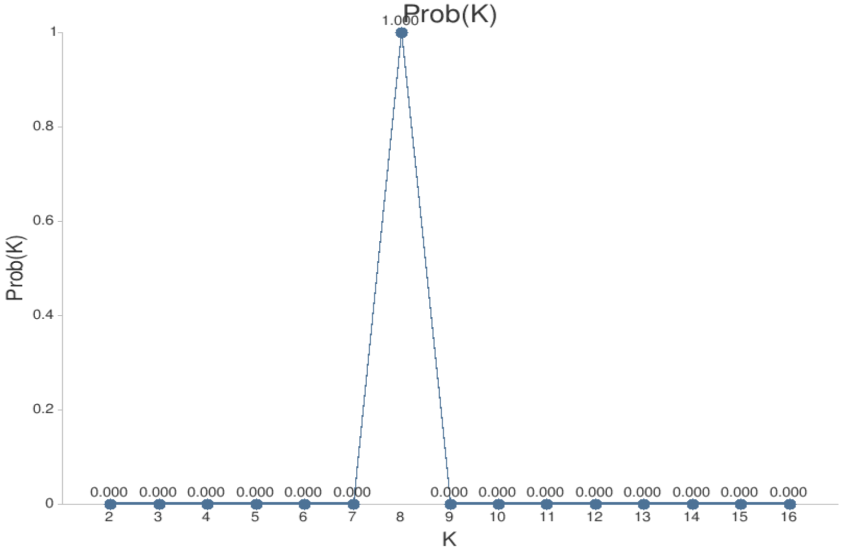

Supplementary Figure 2. Highest K value by Pitchard

The graph shows the highest K value equal to 8 according to probability mesured by Pitchard method.

Supplementary Figure 3. CLUMPAK main pipeline - Job 1704282543 summary

Distruct output plots:

K=2

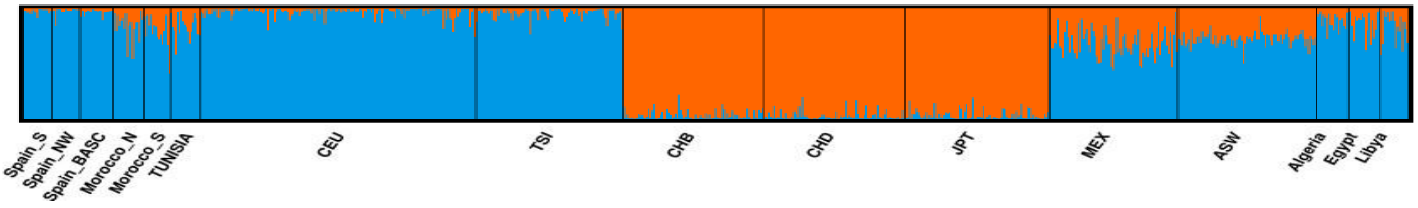

K=3

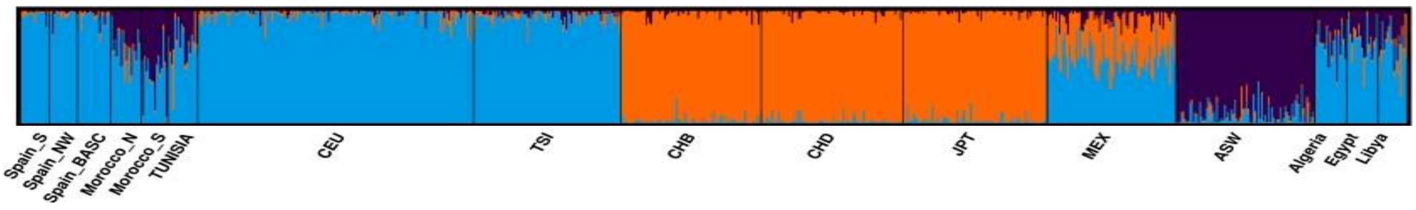

K=4

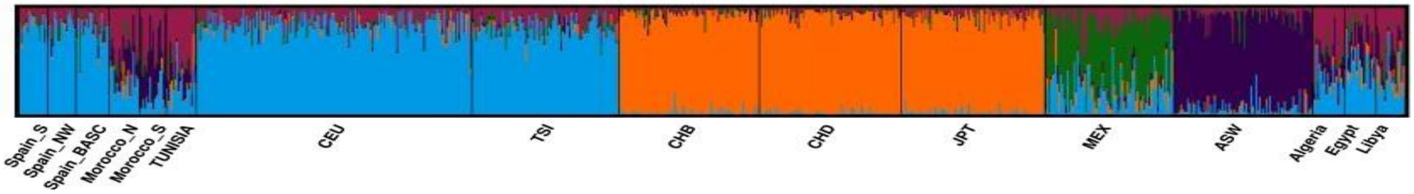
Spain_S
Spain_NW
Spain_BASC
Morocco_N
Morocco_S
TUNISIA
CEU
TSI
CHB
CHD
JPT
MEX
ASW
Algeria
Egypt
Libya

K=5

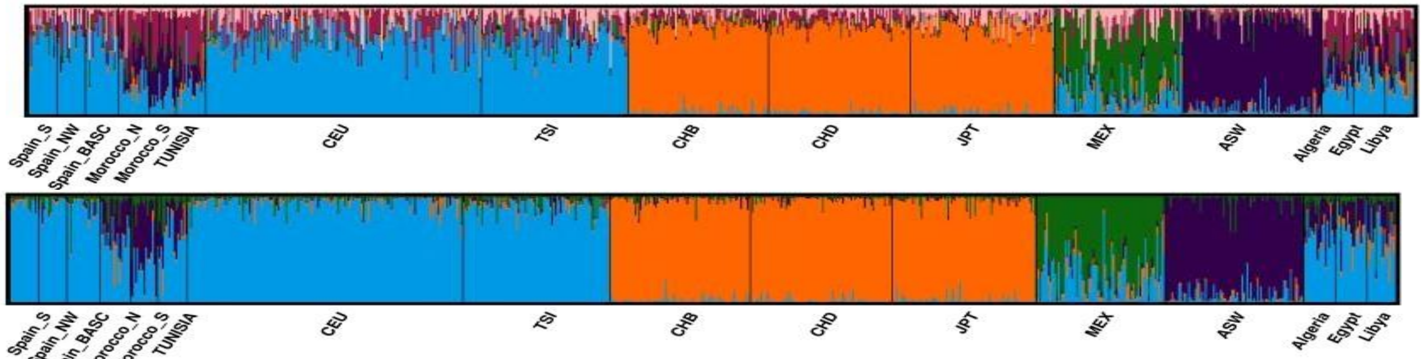
Spain_S
Spain_NW
Spain_BASC
Morocco_N
Morocco_S
TUNISIA
CEU
TSI
CHB
CHD
JPT
MEX
ASW
Algeria
Egypt
Libya

K=7

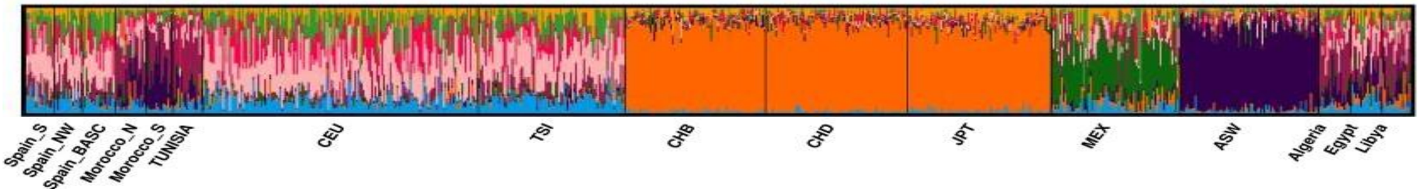
Spain_S
Spain_NW
Spain_BASC
Morocco_N
Morocco_S
TUNISIA
CEU
TSI
CHB
CHD
JPT
MEX
ASW
Algeria
Egypt
Libya

K=8

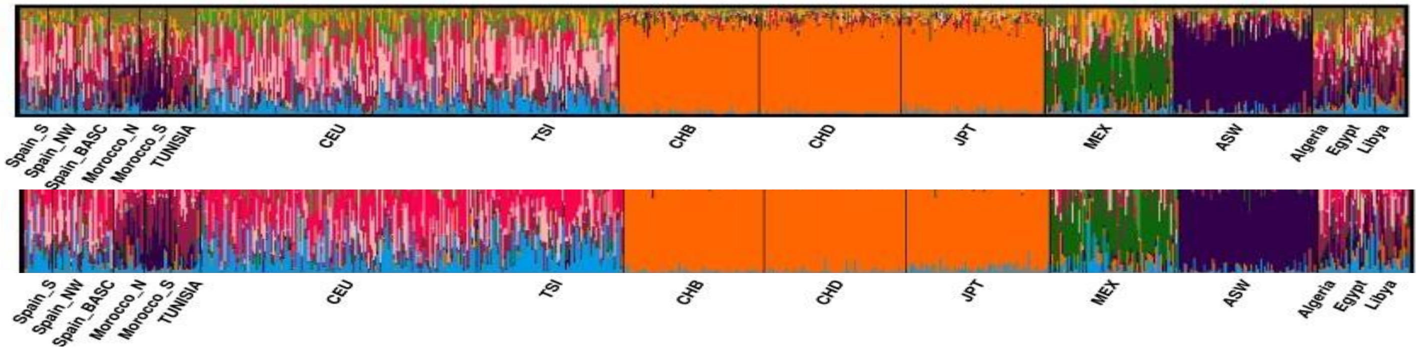
Spain_S
Spain_NW
Spain_BASC
Morocco_N
Morocco_S
TUNISIA
CEU
TSI
CHB
CHD
JPT
MEX
ASW
Algeria
Egypt
Libya

K=10

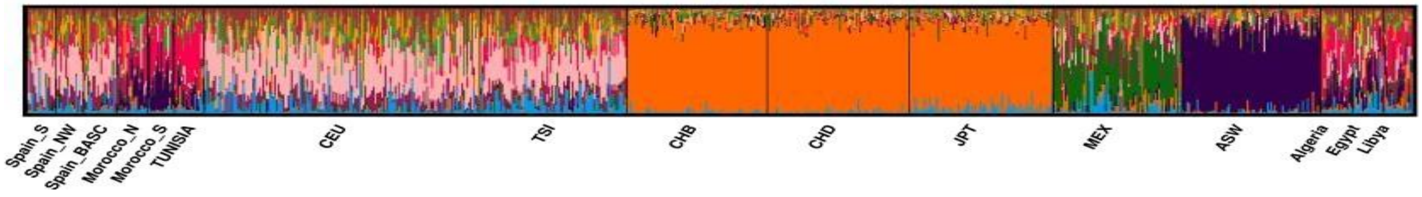

K=11

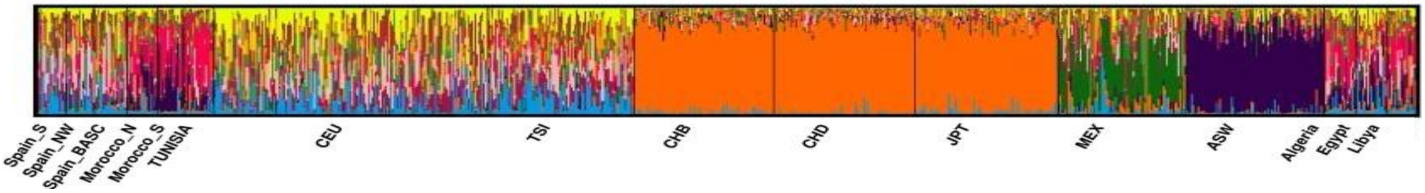

K=12

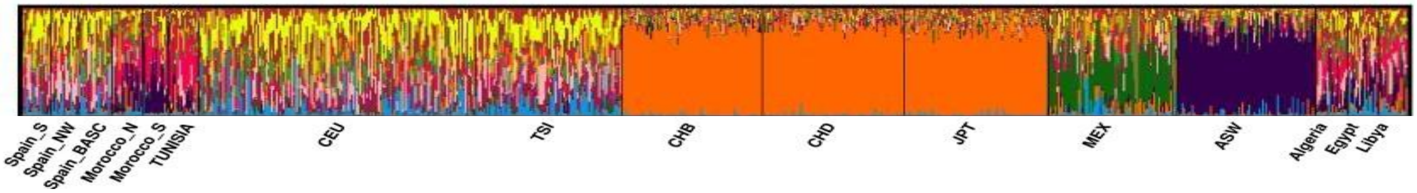

K=13

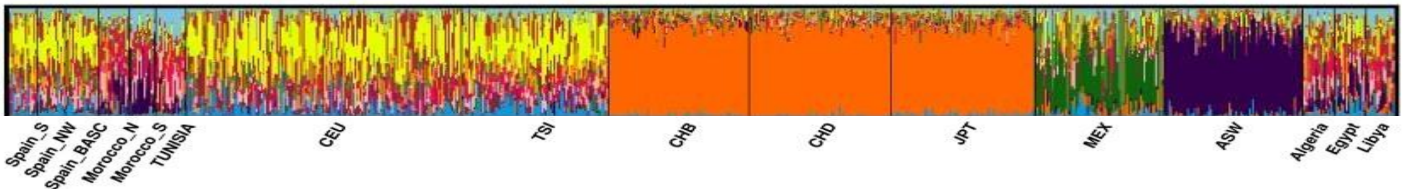

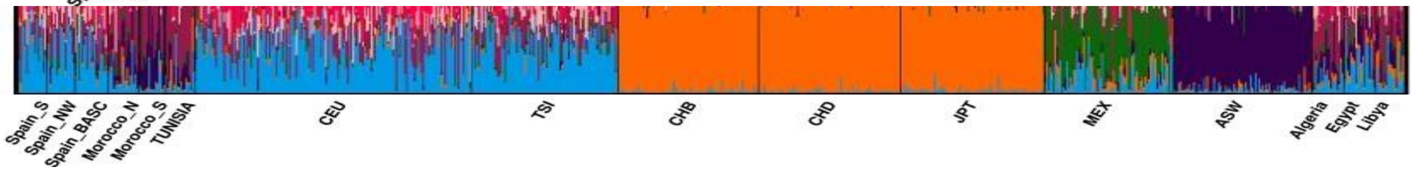

K=15

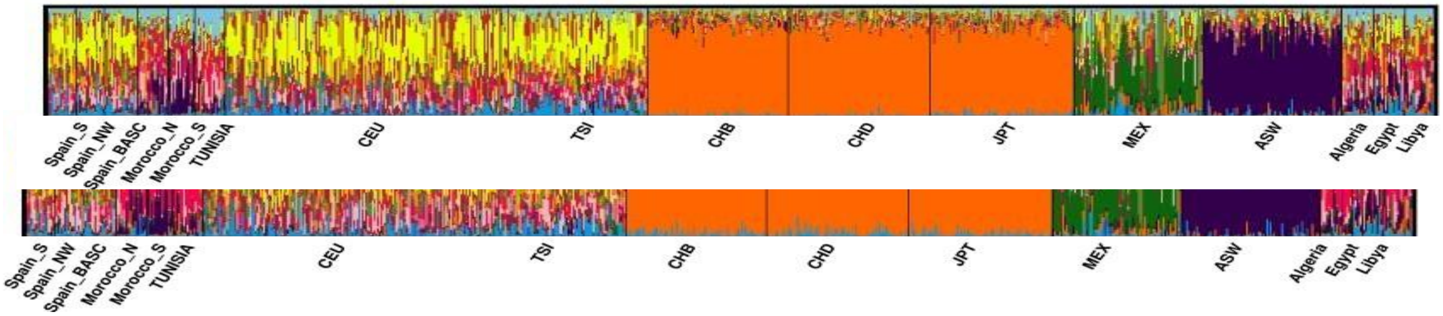
Spain_S
Spain_NW
Spain_BASC
Morocco_N
Morocco_S
TUNISIA
CEU
TSI
CHB
CHD
JPT
MEX
ASW
Algeria
Egypt
Libya

Supplementary Table 1. Summary of studied populations

**Cluster**	**MED**												**AMR**		**EAS**		
**Population**	Algeria	Egypt	Libya	South Morocco	North Morocco	Tunisia	Northwestern and Western European ancestry	Toscani	South Spain (Spain_S	North West of Spain	Spain Basic	African ancestry in the South Western USA	Mexican ancestry living in Los Angeles, California	Han Chinese in Beijing, China	Chinese population of metropolitan Denver, Colorado, USA	Japanese in Tokyo
**Symbol**	ALG	EGY	Libya	Morocco_S	Morocco_N	Tunisia	CEU	TSI	Spain_S	SpainNW	Spain_BASC	ASW	MEX	CHB	CHD	JPT
**Individual Number**	19	19	17	16	18	18	165	88	17	17	20	83	77	84	85	86

Supplementary Table 2. Prisma included studies

**PMID**	**Title**	**Authors**	**Citation**
35819423	Effect of Pharmacogenomic Testing for Drug-Gene Interactions on Medication Selection and Remission of Symptoms in Major Depressive Disorder: The PRIME Care Randomized Clinical Trial	Oslin DW, Lynch KG, Shih MC, Ingram EP, Wray LO, Chapman SR, Kranzler HR, Gelernter J, Pyne JM, Stone A, DuVall SL, Lehmann LS, Thase ME	JAMA, 2022 Jul
32081407	Pharmacogenomics in pregnancy	Betcher HK, George AL Jr.	Semin Perinatol. 2020 Apr
21999760	Opioid pharmacokinetic drug-drug interactions	Overholser BR, Foster DR.	Am J Manag Care. 2011 Sep
28699646	Review of Opioid Pharmacogenetics and Considerations for Pain Management	Owusu Obeng A, Hamadeh I, Smith M.	Pharmacotherapy. 2017 Sep
33728947	Pharmacogenomics of oxycodone: a narrative literature review	Umukoro NN, Aruldhas BW, Rossos R, Pawale D, Renschler JS, Sadhasivam S.	Pharmacogenomics. 2021 Apr

PMID	Title	Authors	Citation
33387367	Clinical Pharmacogenetics Implementation Consortium Guideline for CYP2D6, OPRM1, and COMT Genotypes and Select Opioid Therapy	Crews KR, Monte AA, Huddart R, Caudle KE, Kharasch ED, Gaedigk A, Dunnenberger HM, Leeder JS, Callaghan JT, Samer CF, Klein TE, Haidar CE, Van Driest SL, Ruano G, Sangkuhl K, Cavallari LH, Müller DJ, Prows CA, Nagy M, Somogyi AA, Skaar TC.	Clin Pharmacol Ther. 2021 Oct
33847389	Pharmacogenomics and Morphine	Ofoegbu A, B Ettienne E.	J Clin Pharmacol. 2021 Sep
27861439	Trends in Tramadol: Pharmacology, Metabolism, and Misuse	Miotto K, Cho AK, Khalil MA, Blanco K, Sasaki JD, Rawson R.	Anesth Analg. 2017 Jan
29249361	Pharmacogenomics of GPCR Drug Targets	Hauser AS, Chavali S, Masuho I, Jahn LJ, Martemyanov KA, Gloriam DE, Babu MM.	Cell. 2018 Jan 11
26940689	Are strong opioids equally effective and safe in the treatment of chronic cancer pain? A multicenter randomized phase IV 'real life' trial on the variability of response to opioids	Corli O, Floriani I, Roberto A, Montanari M, Galli F, Greco MT, Caraceni A, Kaasa S, Dragani TA, Azzarello G, Luzzani M, Cavanna L, Bandieri E, Gamucci T, Lipari G, Di Gregorio R, Valenti D, Reale C, Pavesi L, Iorno V, Crispino C, Pacchioni M, Apolone G	
21900886	Pharmacogenetics of naltrexone in asian americans: a randomized placebo-controlled laboratory study	Ray LA, Bujarski S, Chin PF, Miotto K.	Neuropsychopharmacology. 2012 Jan
22713632	Aversive and reinforcing opioid effects: a pharmacogenomic twin study	Angst MS, Lazzeroni LC, Phillips NG, Drover DR, Tingle M, Ray A, Swan GE, Clark JD.	Anesthesiology. 2012 Jul
25558980	Novel associations between FAAH genetic variants and postoperative central opioid-related adverse effects	Sadhasivam S, Zhang X, Chidambaran V, Mavi J, Pilipenko V, Mersha TB, Meller J, Kaufman KM, Martin LJ, McAuliffe J.	Pharmacogenomics J. 2015 Oct

PMID	Title	Authors	Citation
18250251	An evaluation of mu-opioid receptor (OPRM1) as a predictor of naltrexone response in the treatment of alcohol dependence: results from the Combined Pharmacotherapies and Behavioral Interventions for Alcohol Dependence (COMBINE) study	Anton RF, Oroszi G, O'Malley S, Couper D, Swift R, Pettinati H, Goldman D.	Arch Gen Psychiatry. 2008 Feb
34381173	Epigenetic moderators of naltrexone efficacy in reducing heavy drinking in Alcohol Use Disorder: a randomized trial	Schacht JP, Hoffman M, Chen BH, Anton RF.	Pharmacogenomics J. 2022 Feb
36942908	Association of OPRM1, MIR23B, and MIR107 genetic variability with acute pain, chronic pain and adverse effects after postoperative tramadol and paracetamol treatment in breast cancer	Vidic Z, Goricar K, Strazisar B, Besic N, Dolzan V.	Radiol Oncol. 2023 Mar 22
30796320	Polymorphism A118G of opioid receptor mu 1 (OPRM1) is associated with emergence of suicidal ideation at antidepressant onset in a large naturalistic cohort of depressed outpatients	Nobile B, Ramoz N, Jaussent I, Gorwood P, Olié E, Castroman JL, Guillaume S, Courtet P.	Sci Rep. 2019 Feb 22
33420349	A hybrid implementation-effectiveness randomized trial of CYP2D6-guided postoperative pain management	Thomas CD, Parvataneni HK, Gray CF, Deen JT, Prieto HA, Pulido LF, Elsey AR, Elwood EN, Starostik P, Gong Y, Fillingim RB, Johnson JA, Cavallari LH.	Genet Med. 2021 Apr
23934621	The effects of alcohol on the pharmacokinetics and pharmacodynamics of the selective mu-opioid receptor antagonist GSK1521498 in healthy subjects	Ziauddeen H, Nathan PJ, Dodds C, Maltby K, Miller SR, Waterworth D, Song K, Warren L, Hosking L, Zucchetto M, Bush M, Johnson LV, Sarai B, Mogg K, Bradley BP, Richards DB, Fletcher PC, Bullmore ET.	J Clin Pharmacol. 2013 Oct

PMID	Title	Authors	Citation
22444188	Pain sensitivity and opioid analgesia: a pharmacogenomic twin study	Angst MS, Phillips NG, Drover DR, Tingle M, Ray A, Swan GE, Lazzeroni LC, Clark DJ.	Pain. 2012 Jul
29265379	Pharmacogenetic Effects of Naltrexone in Individuals of East Asian Descent: Human Laboratory Findings from a Randomized Trial	Ray LA, Green R, Roche DJO, Bujarski S, Hartwell EE, Lim AC, Rohrbaugh T, Ghahremani D, Hutchison K, Miotto K.	Alcohol Clin Exp Res. 2018 Mar
22429255	Understanding naltrexone mechanism of action and pharmacogenetics in Asian Americans via behavioral economics: a preliminary study	Bujarski S, MacKillop J, Ray LA.	Exp Clin Psychopharmacol. 2012 Jun
30784356	Drug-gene and drug-drug interactions associated with tramadol and codeine therapy in the INGENIOUS trial	Fulton CR, Zang Y, Desta Z, Rosenman MB, Holmes AM, Decker BS, Zhang Y, T Callaghan J, Pratt VM, Levy KD, Gufford BT, Dexter PR, Skaar TC, Eadon MT.	Pharmacogenomics. 2019 Apr
28409564	Predictors of Naltrexone Response in a Randomized Trial: Reward-Related Brain Activation, OPRM1 Genotype, and Smoking Status	Schacht JP, Randall PK, Latham PK, Voronin KE, Book SW, Myrick H, Anton RF.	Neuropsychopharmacology. 2017 Dec
32803728	Effects of Good Pain Management (GPM) ward program on patterns of care and pain control in patients with cancer pain in Taiwan	Su WC, Chuang CH, Chen FM, Tsai HL, Huang CW, Chang TK, Hou MF, Wang JY.	Support Care Cancer. 2021 Apr
29535047	Design and rational for the precision medicine guided treatment for cancer pain pragmatic clinical trial	Mosley SA, Hicks JK, Portman DG, Donovan KA, Gopalan P, Schmit J, Starr J, Silver N, Gong Y, Langaee T, Clare-Salzler M, Starostik P, Chang YD, Rajasekhara S, Smith JE, Soares HP, George TJ Jr, McLeod HL, Cavallari LH.	Contemp Clin Trials. 2018 May
31160146	Neuroimaging findings from an experimental pharmacology trial of	Lim AC, Ghahremani DG, Grodin EN, Green R, Bujarski S, Hartwell EE,	Drug Alcohol Depend. 2019 Jul 1

PMID	Title	Authors	Citation
	naltrexone in heavy drinkers of East Asian descent	Courtney KE, Hutchison K, Miotto K, Ray LA.	
32920647	A Delta-Opioid Receptor Gene Polymorphism Moderates the Therapeutic Response to Extended-Release Buprenorphine in Opioid Use Disorder	Kranzler HR, Lynch KG, Crist RC, Hartwell E, Le Moigne A, Laffont CM, Andorn AC.	Int J Neuropsychopharmacol. 2021 Feb 15
30374191	Opioid system modulation with buprenorphine/samidorphan combination for major depressive disorder: two randomized controlled studies	Fava M, Thase ME, Trivedi MH, Ehrich E, Martin WF, Memisoglu A, Nangia N, Stanford AD, Yu M, Pathak S.	Mol Psychiatry. 2020 Jul
30670877	CYP2D6-guided opioid therapy improves pain control in CYP2D6 intermediate and poor metabolizers: a pragmatic clinical trial	Smith DM, Weitzel KW, Elsey AR, Langaee T, Gong Y, Wake DT, Duong BQ, Hagen M, Harle CA, Mercado E, Nagoshi Y, Newsom K, Wright A, Rosenberg EI, Starostik P, Clare-Salzler MJ, Schmidt SO, Fillingim RB, Johnson JA, Cavallari LH.	Genet Med. 2019 Aug
27286724	Population Genetic-Based Pharmacokinetic Modeling of Methadone and its Relationship with the QTc Interval in Opioid-Dependent Patients	Csajka C, Crettol S, Guidi M, Eap CB.	Clin Pharmacokinet. 2016 Dec
24724887	Pharmacogenetics of naltrexone and disulfiram in alcohol dependent, dually diagnosed veterans	Arias AJ, Gelernter J, Gueorguieva R, Ralevski E, Petrakis IL.	Am J Addict. 2014 May-Jun
26389554	Methadone Pharmacogenetics: CYP2B6 Polymorphisms Determine Plasma Concentrations, Clearance, and Metabolism	Kharasch ED, Regina KJ, Blood J, Friedel C.	Anesthesiology. 2015 Nov

PMID	Title	Authors	Citation
25155932	ABCC3 and OCT1 genotypes influence pharmacokinetics of morphine in children	Venkatasubramanian R, Fukuda T, Niu J, Mizuno T, Chidambaran V, Vinks AA, Sadhasivam S.	Pharmacogenomics. 2014 Jul
21062464	Trial Protocol: Using genotype to tailor prescribing of nicotine replacement therapy: a randomised controlled trial assessing impact of communication upon adherence	Marteau TM, Munafò MR, Aveyard P, Hill C, Whitwell S, Willis TA, Crockett RA, Hollands GJ, Johnstone EC, Wright AJ, Prevost AT, Armstrong D, Sutton S, Kinmonth AL.	BMC Public Health. 2010 Nov 9
31087723	Pharmacogenetic role of dopamine transporter (SLC6A3) variation on response to disulfiram treatment for cocaine addiction	Kampangkaew JP, Spellicy CJ, Nielsen EM, Harding MJ, Ye A, Hamon SC, Kosten TR, Nielsen DA.	Am J Addict. 2019 Jul
18403122	Genetic variability of the mu-opioid receptor influences intrathecal fentanyl analgesia requirements in laboring women	Landau R, Kern C, Columb MO, Smiley RM, Blouin JL.	Pain. 2008 Sep 30
22784013	Variation in OPRM1 moderates the effect of desire to drink on subsequent drinking and its attenuation by naltrexone treatment	Kranzler HR, Armeli S, Covault J, Tennen H.	Addict Biol. 2013 Jan
22509402	Effect on adherence to nicotine replacement therapy of informing smokers their dose is determined by their genotype: a randomised controlled trial	Marteau TM, Aveyard P, Munafò MR, Prevost AT, Hollands GJ, Armstrong D, Sutton S, Hill C, Johnstone E, Kinmonth AL.	PLoS One. 2012
30115121	Topiramate versus naltrexone for alcohol use disorder: study protocol for a genotype-stratified, double-blind randomised controlled trial (TOP study)	Morley KC, Kranzler HR, Luquin N, Baillie A, Shanahan M, Trent R, Teesson M, Haber PS.	Trials. 2018 Aug 16
28244808	CYP2D6 pharmacogenetic and oxycodone pharmacokinetic association study in pediatric surgical patients	Balyan R, Mecoli M, Venkatasubramanian R, Chidambaran V, Kamos N, Clay S, Moore DL, Mavi J, Glover CD, Szmuk P, Vinks A, Sadhasivam S.	Pharmacogenomics. 2017 Mar

PMID	Title	Authors	Citation
12453926	Pharmacogenetics of codeine metabolism in an urban population of children and its implications for analgesic reliability	Williams DG, Patel A, Howard RF.	Br J Anaesth. 2002 Dec
37445931	Pharmacogenetic Guided Opioid Therapy Improves Chronic Pain Outcomes and Comorbid Mental Health: A Randomized, Double-Blind, Controlled Study	Agulló L, Aguado I, Muriel J, Margarit C, Gómez A, Escorial M, Sánchez A, Fernández A, Peiró AM.	Int J Mol Sci. 2023 Jun 28
23635803	ANKK1 and DRD2 pharmacogenetics of disulfiram treatment for cocaine abuse	Spellicy CJ, Kosten TR, Hamon SC, Harding MJ, Nielsen DA.	Pharmacogenet Genomics. 2013 Jul
11318772	A single dose of methadone inhibits cytochrome P-4503A activity in healthy volunteers as assessed by the urinary cortisol ratio	Boulton DW, Arnaud P, DeVane CL.	Br J Clin Pharmacol. 2001 Apr
28637741	The CLOSED trial		
27109624	Opioid Antagonists and the A118G Polymorphism in the μ-Opioid Receptor Gene: Effects of GSK1521498 and Naltrexone in Healthy Drinkers Stratified by OPRM1 Genotype	Ziauddeen H, Nestor LJ, Subramaniam N, Dodds C, Nathan PJ, Miller SR, Sarai BK, Maltby K, Fernando D, Warren L, Hosking LK, Waterworth D, Korzeniowska A, Win B, Richards DB, Vasist Johnson L, Fletcher PC, Bullmore ET.	Neuropsychopharmacology. 2016 Oct
31806881	OPRM1, OPRK1, and COMT genetic polymorphisms associated with opioid effects on experimental pain: a randomized, double-blind, placebo-controlled study	Ho KWD, Wallace MR, Staud R, Fillingim RB.	Pharmacogenomics J. 2020 Jun
32772383	Opioid and Dopamine Genes Interact to Predict Naltrexone Response in a	Anton RF, Voronin KE, Book SW, Latham PK, Randall PK, Glen WB, Hoffman M, Schacht JP.	Alcohol Clin Exp Res. 2020 Oct

PMID	Title	Authors	Citation
	Randomized Alcohol Use Disorder Clinical Trial		

Supplementary Table 3. Pathway Enrichment Analyseis

Category	GO	Go-term	p-value	number of genes
GOTERM_BP_DIRECT	GO:0006805	xenobiotic metabolic process	6.023204611239894E-16	13
GOTERM_BP_DIRECT	GO:0007210	serotonin receptor signaling pathway	4.308385330705686E-15	7
GOTERM_BP_DIRECT	GO:0008210	estrogen metabolic process	3.3945426412321174E-14	9
GOTERM_BP_DIRECT	GO:0060079	excitatory postsynaptic potential	1.3116645492262196E-13	11
GOTERM_BP_DIRECT	GO:0008202	steroid metabolic process	3.6226813879482314E-12	9
GOTERM_BP_DIRECT	GO:0007268	synaptic transmission	1.3029011085374458E-11	13
GOTERM_BP_DIRECT	GO:0042178	xenobiotic catabolic process	5.598499385365412E-9	6
GOTERM_BP_DIRECT	GO:0050877	neurological system process	9.999510679444327E-9	7
GOTERM_BP_DIRECT	GO:0007187	G-protein coupled receptor signaling pathway, coupled to cyclic nucleotide second messenger	1.026584159142599E-9	7
GOTERM_BP_DIRECT	GO:0007200	phospholipase C-activating G-protein coupled receptor signaling pathway	6.38038118523233E-8	7
GOTERM_BP_DIRECT	GO:0016098	monoterpenoid metabolic process	5.344509573836911E-7	4
GOTERM_BP_DIRECT	GO:0042391	regulation of membrane potential	5.898931848715895E-7	7
GOTERM_BP_DIRECT	GO:0098662	inorganic cation transmembrane transport	1.798272948745557E-7	5
GOTERM_BP_DIRECT	GO:0009410	response to xenobiotic stimulus	9.229886648996538E-7	9
GOTERM_BP_DIRECT	GO:0034220	ion transmembrane transport	3.888747617744638E-6	7

Category	GO	Go-term	p-value	number of genes
GOTERM_BP_DIRECT	GO:0070374	positive regulation of ERK1 and ERK2 cascade	5.488860272481066E-6	8
GOTERM_BP_DIRECT	GO:0019373	epoxygenase P450 pathway	2.9552010051053808E-5	4
GOTERM_BP_DIRECT	GO:0009822	alkaloid catabolic process	2.7346018331947907E-5	3
GOTERM_BP_DIRECT	GO:0070989	oxidative demethylation	1.1923509280524878E-5	4
GOTERM_BP_DIRECT	GO:0051209	release of sequestered calcium ion into cytosol	1.8891395551643763E-5	5
GOTERM_BP_DIRECT	GO:0042755	eating behavior	9.289194261798303E-5	4
GOTERM_BP_DIRECT	GO:0050955	thermoception	5.458468110414458E-5	3
GOTERM_BP_DIRECT	GO:0019233	sensory perception of pain	1.8891395551643763E-5	5
GOTERM_BP_DIRECT	GO:0043278	response to morphine	5.20146945719239E-5	4
GOTERM_BP_DIRECT	GO:0051930	regulation of sensory perception of pain	1.13777786950086E-4	4
GOTERM_BP_DIRECT	GO:0038003	opioid receptor signaling pathway	2.5273538762824376E-4	3
GOTERM_BP_DIRECT	GO:0001666	response to hypoxia	1.26572481787592E-4	6
GOTERM_BP_DIRECT	GO:0006082	organic acid metabolic process	1.13777786950086E-4	4
GOTERM_BP_DIRECT	GO:0045907	positive regulation of vasoconstriction	1.13777786950086E-4	4
GOTERM_BP_DIRECT	GO:0035094	response to nicotine	1.9413599644553195E-4	4
KEGG_PATHWAY	hsa04726	Serotonergic synapse	2.416661606075805E-16	16
KEGG_PATHWAY	hsa00982	Drug metabolism - cytochrome P450	2.0496611403446925E-11	11
KEGG_PATHWAY	hsa00980	Metabolism of xenobiotics by cytochrome P450	4.673241052245618E-11	11
KEGG_PATHWAY	hsa05204	Chemical carcinogenesis - DNA adducts	1.0826076922649092E-8	9
KEGG_PATHWAY	hsa04080	Neuroactive ligand-receptor interaction	4.906124688794852E-8	15

Category	GO	Go-term	p-value	number of genes
KEGG_PATHWAY	hsa00140	Steroid hormone biosynthesis	2.5320764260241253E-6	7
KEGG_PATHWAY	hsa00830	Retinol metabolism	4.376992318800413E-6	7
KEGG_PATHWAY	hsa04976	Bile secretion	2.0954813800631094E-5	7
KEGG_PATHWAY	hsa05207	Chemical carcinogenesis - receptor activation	5.6722019196236984E-5	9
KEGG_PATHWAY	hsa04742	Taste transduction	2.174310982563563E-4	6
KEGG_PATHWAY	hsa05321	Inflammatory bowel disease	7.850600272763479E-4	5
KEGG_PATHWAY	hsa01523	Antifolate resistance	9.135085647933975E-4	4
UP_KW_BIOLOGICAL_PROCESS	KW-0753	Steroid metabolism	5.067751961317711E-9	9
UP_KW_BIOLOGICAL_PROCESS	KW-0085	Behavior	4.615367305097824E-6	4
UP_KW_BIOLOGICAL_PROCESS	KW-0406	Ion transport	5.421628769811925E-6	13
UP_KW_BIOLOGICAL_PROCESS	KW-0443	Lipid metabolism	1.8399442428564817E-5	13
BIOCARTA	h_nuclearRsPathway	Nuclear Receptors in Lipid Metabolism and Toxicity	2.094043157401423E-4	5
